# The cost-effectiveness of oral HIV pre-exposure prophylaxis and early antiretroviral therapy in the presence of drug resistance among men who have sex with men in San Francisco

**DOI:** 10.1186/s12916-018-1047-1

**Published:** 2018-04-24

**Authors:** Mingwang Shen, Yanni Xiao, Libin Rong, Lauren Ancel Meyers, Steven E. Bellan

**Affiliations:** 1 Department of Epidemiology and Biostatistics, School of Public Health, Xi’an Jiaotong University Health Science Center, Xi’an, Shaanxi 710061 People’s Republic of China; 2 School of Mathematics and Statistics, Xi’an Jiaotong University, Xi’an, 710049 People’s Republic of China; 3 Department of Integrative Biology, The University of Texas at Austin, Austin, TX 78712 USA; 4 Department of Mathematics, University of Florida, Gainesville, FL 32611 USA; 5 The Santa Fe Institute, Santa Fe, NM 87501 USA; 6 Department of Epidemiology and Biostatistics, College of Public Health, University of Georgia, Athens, GA 30602 USA; 7 Center for Ecology of Infectious Diseases, University of Georgia, Athens, GA 30602 USA

**Keywords:** Cost-effectiveness, Pre-exposure prophylaxis, Earlier ART initiation, Drug resistance, Mathematical model

## Abstract

**Background:**

Poor adherence to either antiretroviral treatment (ART) or pre-exposure prophylaxis (PrEP) can promote drug resistance, though this risk is thought to be considerably higher for ART. In the population of men who have sex with men (MSM) in San Francisco, PrEP coverage reached 9.6% in 2014 and has continued to rise. Given the risk of drug resistance and high cost of second-line drugs, the costs and benefits of initiating ART earlier while expanding PrEP coverage remain unclear.

**Methods:**

We develop an infection–age-structured mathematical model and fit this model to the annual incidence of AIDS cases and deaths directly, and to resistance and demographic data indirectly. We investigate the impact of six various intervention scenarios (low, medium, or high PrEP coverage, with or without earlier ART) over the next 20 years.

**Results:**

Low (medium, high) PrEP coverage with earlier ART could prevent 22% (42%, 57%) of a projected 44,508 total new infections and 8% (26%, 41%) of a projected 18,426 new drug-resistant infections, and result in a gain of 43,649 (74,048, 103,270) QALYs over 20 years compared to the status quo, at a cost of $4745 ($78,811, $115,320) per QALY gained, respectively.

**Conclusions:**

High PrEP coverage with earlier ART is expected to provide the greatest benefit but also entail the highest costs among the strategies considered. This strategy is cost-effective for the San Francisco MSM population, even considering the acquisition and transmission of ART-mediated drug resistance. However, without a substantial increase to San Francisco’s annual HIV budget, the most advisable strategy may be initiating ART earlier, while maintaining current strategies of PrEP enrollment.

**Electronic supplementary material:**

The online version of this article (10.1186/s12916-018-1047-1) contains supplementary material, which is available to authorized users.

## Background

In July 2012, the US Food and Drug Administration (FDA) approved tenofovir/emtricitabine for use as oral pre-exposure prophylaxis (PrEP) [1]. Estimates of PrEP efficacy in preventing HIV infection range from 39% to 86% in randomized controlled trials [2–6]. PrEP consumer demand has accelerated since mid-2013 [7]. PrEP coverage among men who have sex with men (MSM) in San Francisco was estimated at 9.6% in 2014 [8]. In May 2016, more than 6000 MSM in San Francisco were reported to receive PrEP [9], suggesting a coverage of approximately 12% given that the HIV-negative MSM population is estimated at 50,000 [7, 10, 11]. In 2010, prior to this increased PrEP uptake, San Francisco was one of the first cities to institute guidelines to initiate antiretroviral therapy (ART) as early as possible post-infection rather than waiting for signs of disease progression, such as clinical symptoms or low CD4+ cell counts [12], given findings that early ART initiation improves survival while reducing the risks of transmission to others [13]. However, it has been hypothesized that early ART initiation might provide more time for the evolution of drug resistance and that subsequent transmission of drug-resistant HIV might reduce PrEP effectiveness [14–16].

Herein, we address the long-term population-level costs and health benefits of expanding PrEP coverage in combination with increasingly early ART initiation among the MSM population in San Francisco. We assess the effectiveness and cost-effectiveness of expanding PrEP coverage, with and without earlier ART guidelines, using an infection–age-structured model [17, 18]. The model tracks the transmission rate and life expectancy of individuals at each infection age, while accounting for ART-mediated drug resistance and the costs of treatment failure and second-line regimens. We fit this model to epidemiological data on the annual incidence of newly diagnosed AIDS cases and deaths amongst MSM in San Francisco (simultaneously matching prevalence, resistance, and demographic data [18]) and estimate the impact of various intervention scenarios over the next 20 years.

Several modeling studies have addressed the impact of PrEP in San Francisco, including estimates of coverage required to curtail transmission [7], an assessment of whether PrEP might increase the drug-resistant transmission [14], and a cost-effectiveness analysis of PrEP in combination with a partially effective HIV vaccine [19]. Our study differs from these prior analyses and others of PrEP cost-effectiveness [20–26] in the United States in several aspects. First, we analyze the expansion in PrEP coverage in conjunction with changing ART guidelines, rather than comparing initiation of PrEP to no PrEP [7, 14, 19–24, 27, 28]. Second, we model the acquisition and transmission of ART-mediated resistance and the indirect population-level effects of PrEP (i.e., by reducing the number of infected individuals, PrEP indirectly benefits other individuals in the population who could have otherwise been infected by individuals on PrEP), which have not been addressed in previous studies [7, 19–25]. While two prior analyses of PrEP [16, 26] considered ART-mediated resistance and subsequent treatment failure, one [16] did not address the impact of earlier ART initiation on PrEP cost-effectiveness, and the other [26] did not account for increased costs and effectiveness of second-line drugs. Third, we consider increasingly earlier initiation of ART and the consequent increases in survival. In contrast, many prior studies modeled early ART by assuming individuals initiate ART whilst their CD4+ T cell counts remain above 350 cells/mm^3^ [29], by assuming ART eligibility occurs once their CD4+ T cell count drops below 500 cells/mm^3^ [30, 31], or by assuming ART initiation occurs within 2 years of infection [32]. Two studies that modeled ART initiation at 1 year post-infection [33, 34] did not incorporate the increased survival times, which may cause underestimation of intervention cost-effectiveness.

## Methods

**Fig. 1:**
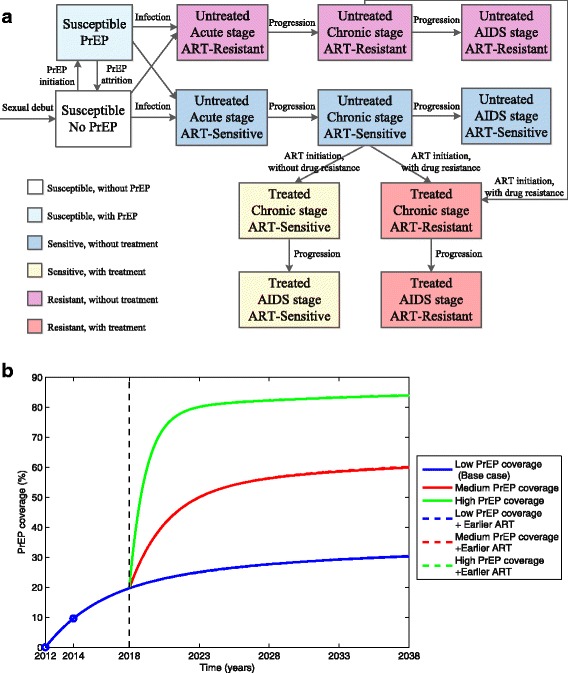
**a** Flow diagram of the HIV transmission dynamics with PrEP and ART interventions, incorporating the acquisition and transmission of drug resistance following ART. **b** Low, medium, and high PrEP coverage scenarios in the San Francisco MSM population. PrEP coverage increased to an estimated 9.6% (blue circle) of San Francisco’s MSM population by 2014 [8] after the Food and Drug Administration approved PrEP in 2012. Projection to 2023 of this rate of increase yields a PrEP coverage of 25% in 2023 (low PrEP scenarios). Efforts to further expand PrEP coverage were modelled by identifying a constant PrEP initiation rate that yielded coverage levels that saturated at 50% (medium PrEP coverage) and 80% (high PrEP coverage) by 2023. Earlier initiation of ART minimally affects PrEP coverage projections because, while it leads to increases in the number of susceptible individuals and, consequently, the numbers of PrEP users (Additional file 1: Figure S2), their ratio (i.e., PrEP coverage) remains relatively unchanged. *PrEP* pre-exposure prophylaxis, *ART* antiretroviral therapy, *MSM* men who have sex with men

We extended our previously developed infection–age-structured model [18] to include a PrEP class (see the schematic diagram of the model structure in Fig. 1a, Additional file 1: Figure S1 and Table S1). We modeled the MSM population in San Francisco aged 18–65 years old [10, 18], assuming that first- and second-line ART reduced infectivity by 96% [13] and 80% [18], respectively. Here, the effectiveness of second-line drugs for drug-resistant cases is assumed to be lower than that of first-line drugs for drug-sensitive cases due to lower adherence [35]. We assume that earlier ART initiation confers longer life expectancy, as indicated previously [18, 36], based on clinical data [37, 38] and that treated individuals with drug resistance have life expectancies that are 11 years (varying from 0 to 20 years in sensitivity analyses) shorter than drug-sensitive patients [18, 37, 38]. We estimate that the average period from infection to ART initiation in San Francisco has been 1.6 years, on average [18] (‘early ART’, see Additional file 1: Supplementary Material for details). We compared early ART to ‘earlier’ ART, i.e., treatment initiation at 1 year post-infection, on average. Of the 33% of treated MSM who are virally unsuppressed [10], 76% exhibit drug resistance [39]; we thus assume that 25% of treated MSM in San Francisco [18] have acquired drug resistance as our base case (varied from 0 to 100% in a sensitivity analysis). Resistance here is assumed to be resistant to one or more first-line ART drugs. Given local monitoring protocols, we assume that drug-resistant cases are quickly switched to second-line drugs. We do not differentiate drug combinations beyond the categorizations of first- or second-line for two reasons. First, data to estimate drug-specific efficacies and adherence levels is not readily available. Second, drug-specific parameters are unnecessary to achieve our goal of estimating intervention cost-effectiveness when using these assumptions regarding the average relative effectiveness of first-line and second-line regimens.

We assumed PrEP effectiveness against drug-sensitive strains was 53% based on a meta-analysis [40], where this value reflects both biomedical efficacy and adherence. We assumed relative PrEP effectiveness against resistant strains was 50% (the ratio of PrEP effectiveness against drug-resistant versus drug-sensitive strains) [14–16]. In a sensitivity analysis, we varied PrEP effectiveness against drug-sensitive strains from 10% to 90% [21, 27] and relative effectiveness against drug-resistant strains from 0 to 1. We assumed an 8% annual rate of PrEP attrition, based on a cohort study in San Francisco [41], and varied it from 1% to 30% per year in sensitivity analyses. Based on low empirically observed dropout proportions [10], we assume that all individuals who initiate ART remain on ART until the end of life and do not drop out of care. We considered only ART-mediated and not PrEP-mediated resistance, because both clinical data [42, 43] and mathematical models [44, 45] suggest that PrEP contributes less than 5% to the total burden of resistance since PrEP-selected resistant phenotypes decay below detection by 6 months after drug cessation and remain undetectable for at least 2 years thereafter [46].

### Model calibration

**Fig. 2:**
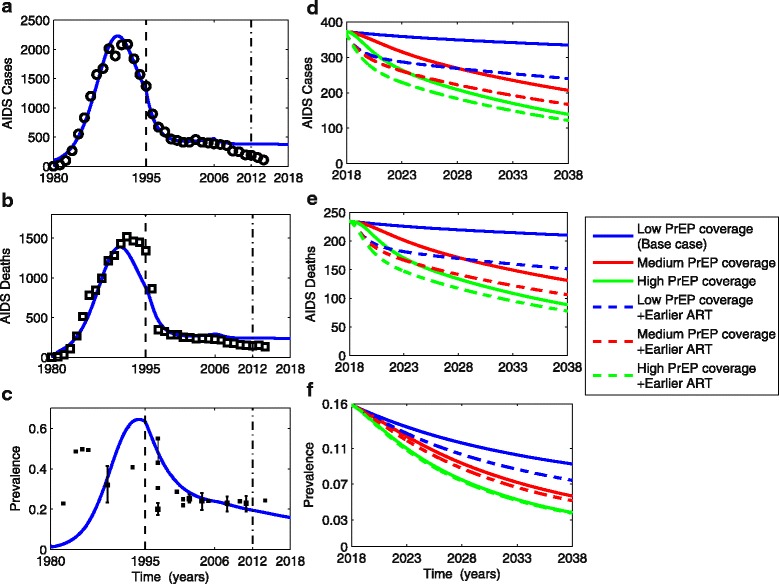
Model fit (blue lines) to observed AIDS incidence (black circles in **a**), AIDS deaths (black hollow squares in **b**) in a San Francisco MSM cohort. **c** Comparing model-generated prevalence with observed HIV prevalence data among sampled MSM populations (black solid squares and 95% confidence interval if available). Dashed vertical black line divides the pre-treatment and post-treatment phases of our model, roughly approximating the increase in ART availability post-1995 in San Francisco. Dot-dashed vertical black lines show rollout of low coverage PrEP starting in 2012. AIDS cases (**d**), deaths (**e**), and prevalence (**f**) are projected for different PrEP coverage levels, with or without earlier ART (as shown in Fig. 1b) over the next 20 years (2018–2038) with the Y-axis rescaled relative to the left panels to clarify the differences between scenarios (blue lines in left and right panels correspond). *PrEP* pre-exposure prophylaxis, *ART* antiretroviral therapy, *MSM* men who have sex with men

To achieve a realistic baseline scenario, we fitted our model to the annual incidence of diagnosed AIDS cases and deaths from 1980 to 2014 among MSM in San Francisco (Fig. 2a–c) using data from the San Francisco Department of Public Health HIV Epidemiology Section (see [18] for details). We modeled five distinct intervention eras, namely (1) no ART availability (1980–1995); (2) ART administered based on clinical symptoms or CD4 thresholds (1995–2006); (3) expanded ART and shortened time to begin ART based on name-based HIV reporting, which, without altering treatment guidelines, increased ART coverage [18, 47] (2006–2012); (4) initial PrEP roll out (2012–2018); and (5) expanded PrEP, with or without earlier ART (2018–2038). We estimated the parameters by fitting the model to data in eras 1–4, and then simulated various intervention scenarios in era 5. The detailed calibration of the first three eras has been previously provided [18] and the parameters of these eras in this study are the same as used therein. We calibrated the model to the fourth era by choosing a constant rate for PrEP initiation such that PrEP coverage rose from 0% in 2012 (post-Food and Drug Administration approval) to the most recent observed value of 9.6% in 2014 [8] (Fig. 1b). At this initiation rate (low PrEP coverage scenario), PrEP coverage would reach 25% (low coverage) after 5 years (2023). For the fifth era, we simulated various intervention scenarios, including continuation of PrEP initiation at this low level or increasing to 50% (medium) or 80% (high) PrEP coverage by 2023. We also considered how these PrEP scenarios interacted with the implementation of new, earlier ART guidelines (1 year post-infection versus the status quo of 1.6 years post-infection). We combined each of the three levels of PrEP intervention with each of the two timings of ART to model six different intervention strategies over the 2018–2038 period (Fig. 1b). The PrEP initiation rates were assumed to be constant for each of the low, medium, and high PrEP scenarios, with rates chosen such that PrEP coverage would saturate at the specified values for each scenario.

**Fig. 3:**
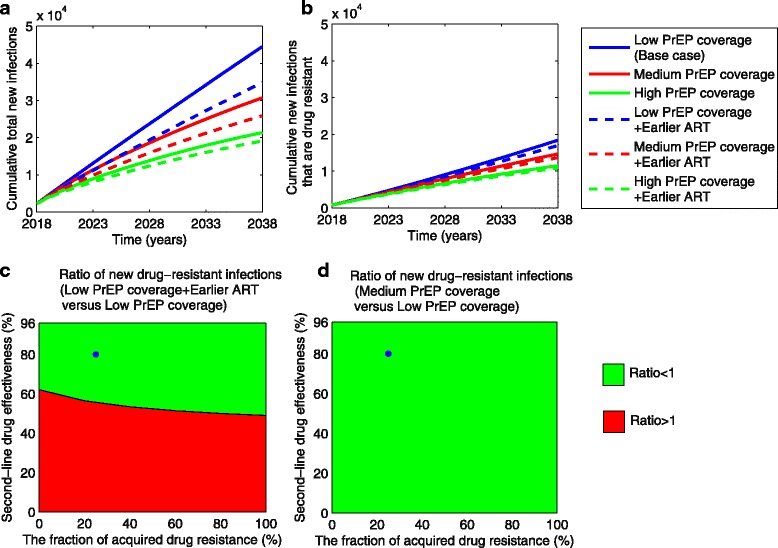
The cumulative total incidence (**a**) and drug-resistant incidence (**b**) from 2018 to 2038 for different combinations of PrEP coverage and ART initiation time (as shown in Fig. 1b). The ratios of cumulative drug-resistant incidence over 20 years for low PrEP coverage plus earlier ART versus the status quo (**c**), and for medium PrEP coverage versus the status quo (**d**), respectively, where we consider the status quo as the low PrEP coverage scenario. The green and red areas in **c** indicate parameter combinations in which adding earlier ART to the status quo would decrease and increase the incidence of drug resistance, respectively. The drug-resistant incidence always decreases for medium PrEP coverage across all range of second-line drug effectiveness as shown in green area in **d**. The blue circles in **c** and **d** denote the base cases (the second-line drug effectiveness is 80% and 25% of treated cases have acquired drug resistance, and all of them switch to second-line drugs timely). All other parameters are fixed as shown in Additional file 1: Table S1. *PrEP* pre-exposure prophylaxis, *ART* antiretroviral therapy

We predicted how the AIDS cases, deaths, prevalence (Fig. 2d–f), total new infections (incidence), and new drug-resistant infections (Fig. 3a, b), as well as the fraction of new infections that are drug resistant (Additoinal file 1: Figure S3) would change after 2018 under each of the six strategies, using the low PrEP and baseline ART strategy as our reference comparator. Additionally, we examined the potential for the incidence of drug-resistant infections to increase as a function of second-line drug effectiveness, PrEP coverage, and ART timing (Fig. 3c, d). All analyses were carried out in *Matlab*.

### Economic model

We used published quality of life estimates for each health state (Additional file 1: Table S1) [23, 26, 48–51]. We assumed that PrEP did not reduce the quality of life [23, 26], but that the quality of life for drug-resistant cases decreases by 5% relative to drug-sensitive individuals at the same stage [26, 52] (varied from 0 to 10% in sensitivity analysis).

We also used published estimates of HIV-related healthcare costs, first- and second-line ART costs, the costs of antibody testing, genotype resistance testing, and counseling and diagnosis [23, 26, 49–51]. Our base case assumed that second-line drug costs for drug-resistant patients were 1.24 times higher than first-line drug costs for drug-sensitive individuals [53], and varied this multiplier from 1 to 5 in sensitivity analysis. Annual PrEP costs included ART medication, laboratory fees (i.e., HIV antibody test every 2–3 months, sexually transmitted infections test every 6 months), and professional fees for patient visits and consultations [54].

We discounted costs and quality-adjusted life years (QALYs) at 3% annually [55] and expressed costs in 2017 U.S. dollars. We calculated net health benefits (QALYs) and costs for various strategies over a 20-year time horizon assuming a public health perspective. The incremental cost-effectiveness ratio (ICER) for each strategy was calculated relative to both the status quo and the next best strategy. Using WHO standards [56], we denoted strategies with an ICER less than the per capita gross domestic product (GDP; $81,347 for San Francisco in 2015 [57]) as very cost-effective and those with an ICER less than three times the per capita GDP as cost-effective ($244,041).

### Sensitivity analysis

We used sensitivity analyses to examine the impact of various model parameters on intervention cost-effectiveness, including the effectiveness of PrEP against drug-sensitive and drug-resistant strains, PrEP coverage, PrEP attrition rate, annual PrEP costs, ART effectiveness, and costs for first- and second-line drug regimens, as well as the reduction in lifespan of treated drug-resistant cases relative to treated drug-sensitive cases. We reported additional sensitivity analyses of ICER estimates to various combinations of PrEP coverage and ART timing in Additional file 1: Supplementary Material.

## Results

### Health outcomes

Under baseline levels of PrEP and ART coverage, we estimate that 44,508 total new HIV infections and 18,426 new drug-resistant infections would occur among MSM in San Francisco over the next 20 years (Table 1 and Fig. 3a, b). Assuming that ART timing remains unchanged, an increase in PrEP coverage to 50% of the MSM population is expected to prevent 13,798 total infections (31% of projected incidence under baseline PrEP and ART) and 3741 drug-resistant infections (20%), and yield 46,757 incremental QALYs (Table 1). An even greater increase in PrEP coverage (to 80%), would avert 23,138 total new infections (52%) and 6939 (38%) new drug-resistant infections, yielding an additional 87,411 QALYs relative to the status quo. Expanding PrEP coverage not only reduces AIDS cases (Fig. 2d) and deaths (Fig. 2e), but also decreases HIV prevalence (Fig. 2f). A 50% (or 80%) PrEP coverage would reduce the prevalence after 20 years from 9.26% in the base scenario to 5.67% (or 3.81%) (Table 1).

**Table 1:** Benefits and costs of expanding PrEP coverage and earlier ART strategies over 20 years

Strategy	Total new Infections^a^	Total infections prevented (fraction)^a^	New infections with drug-resistant strains^a^	Drug-resistant infections prevented (fraction)^a^	HIV prevalence in 2038 (%)	Total costs of PrEP (million US $)^b,c^	Total costs (million US $)^c^	Total QALYs^c^	Incremental costs (million US $)^d^	Incremental QALYs^d^	ICER relative to the status quo	ICER relative to next best strategy
Status quo (Low PrEP coverage)	44,508	–	18,426	–	9.26	5648	9189	1,315,057	–	–	–	–
Medium PrEP coverage	30,710	13,798 (31%)	14,685	3741 (20%)	5.67	11,577	14,666	1,361,814	5477	46,757	117,130	Dominated^e^
High PrEP coverage	21,370	23,138 (52%)	11,487	6939 (38%)	3.81	18,056	20,773	1,402,468	11,584	87,411	132,520	Dominated^e^
Low PrEP coverage+Earlier ART	34,808	9700 (22%)	16,969	1457 (8%)	7.43	5914	9396	1,358,706	207	43,649	4745	4745
Medium PrEP coverage+Earlier ART	25,892	18,616 (42%)	13,686	4740 (26%)	5.14	11,905	15,025	1,389,105	5836	74,048	78,811	185,160
High PrEP coverage+Earlier ART	19,093	25,414 (57%)	10,867	7559 (41%)	3.74	18,307	21,098	1,418,327	11,909	103,270	115,320	207,830

*PrEP* pre-exposure prophylaxis, *ART* antiretroviral therapy, *ICER* incremental cost-effectiveness ratio, *QALY* quality-adjusted life-year

^a^Total new infections, new drug-resistant infections, total HIV infections prevented and drug-resistant infections prevented are undiscounted number from 2018 to 2038

^b^Includes the costs of antiretroviral drugs for PrEP, laboratory fees (such as HIV antibody test fee every 2–3 months, test fee for sexually transmitted infections every 6 months) and professional fees (such as visiting and consulting fees) [54]

^c^Costs and quality-adjusted life years (QALYs) are net present values (3% annual discount rate) over 20 years

^d^Incremental costs and QALYs are relative to the status quo

^e^The strategy that is dominated yields fewer QALYs at higher costs than the comparator and thus is not an effcient use of resources

Relative to the base scenario, combining low or medium PrEP coverage and earlier ART is expected to avert an additional 9700 (22%) or 18,616 (42%) total new infections, 1457 (8%) or 4740 (26%) new drug-resistant infections, and add 43,649 or 74,048 QALYs, respectively. High PrEP coverage combined with earlier ART provides the most health benefits, with an additional 25,414 (57%) total new infections and 7559 (41%) new drug-resistant infections averted relative to the base scenario (Table 1 and Fig. 3a, b). HIV prevalence would drop to 7.43%, 5.14%, and 3.74% after 20 years when earlier ART is combined with low, medium, and high PrEP coverage, respectively.

Figure 3a, b shows that, in the base case, combining earlier ART and expanded PrEP coverage leads to reductions in drug-resistant incidence and even greater reductions in total incidence. For example, adding earlier ART reduces incidence of drug-resistant and total cases by 8% and 22%, respectively, and expansion to medium PrEP coverage leads to respective reductions of 20% and 31% (Table 1). However, the fraction of new infections that are drug resistant is expected to increase across all scenarios (Additional file 1: Figure S3b), in accordance with previous findings [14, 18]. Earlier ART combined with the low levels of PrEP coverage may also increase absolute drug-resistant incidence (i.e., not just the drug-resistant fraction of total incidence) relative to the base case only when second-line drug effectiveness is less than 60% (Fig. 3c). However, expansion to medium or high PrEP coverage decreases drug-resistant incidence (Fig. 3d) across all levels of second-line drug effectiveness both with and without earlier ART.

### Economic outcomes

**Fig. 4:**
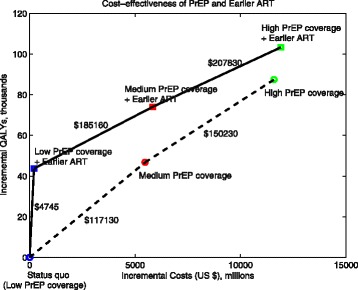
Incremental costs and QALYs of expanded PrEP coverage with and without earlier ART (Fig. 1b), with the origin corresponding to the status quo of low PrEP coverage and baseline ART guidelines. The solid lines show the incremental cost-effectiveness ratio (ICER) relative to the next best strategy. The dashed lines show the ICER relative to the next best strategy when earlier ART is not implemented. These strategies are dominated by similar strategies with earlier ART, meaning that they are not an efficient use of resources. Incremental costs and QALYs are calculated over a 20-year time horizon (2018–2038) and are discounted to the present at 3% annually. *PrEP* pre-exposure prophylaxis, *ART* antiretroviral therapy, *ICER* incremental cost-effectiveness ratio, *QALY* quality-adjusted life-year

Expanding PrEP to either medium or high levels of coverage without changing ART timing was cost-effective by WHO standards [56], with an ICER of $117,130 or $132,520 per QALY gained, respectively, compared to the status quo (Table 1 and Fig. 4). Medium PrEP coverage would cost an additional $5477 million over 20 years ($5929 million for PrEP prevention minus $452 million in treatment savings; Additional file 1: Table S2), or $274 million annually, which is five times San Francisco’s 2015–2016 annual HIV budget ($16.8 million for prevention and $37.6 million for care [58]).

Adding earlier ART to baseline PrEP coverage is the most cost-effective strategy under our base assumptions, costing only $4745 per QALY gained relative to the status quo (Table 1 and Fig. 4). Combining earlier ART with expanded PrEP coverage (50% or 80%) provides more health benefits and costs $78,811 or $115,320 per QALY gained, respectively, compared to the status quo, costing an additional $5836 or $11,909 million, respectively, over 20 years relative to the status quo. However, incremental expansion of PrEP coverage when earlier ART is already in place is substantially more expensive. In particular, earlier ART with medium PrEP coverage costs $185,160 per QALY gained compared to earlier ART with low PrEP coverage level, and earlier ART with high PrEP coverage costs $207,830 per QALY gained compared to earlier ART with medium coverage. This is because earlier ART has already led to a substantial reduction in HIV infections (Fig. 2) and great gains in QALYs (Table 1), such that increased PrEP coverage provides protection only to individuals at a lower infection risk, yielding only modest gains in QALYs per unit cost (Table 1 and Fig. 4).

### Sensitivity analysis

**Fig. 5:**
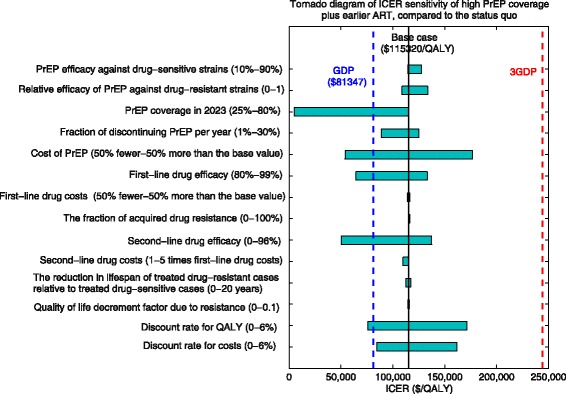
One-way sensitivity analysis of the cost-effectiveness of high PrEP coverage plus earlier ART compared to the status quo. The horizontal bars represent the range of the incremental cost-effectiveness ratios (ICERs) as each variable is varied across its plausible range listed. The solid vertical black line indicates the base case ICER ($115,320 per QALY gained). The dashed vertical blue and red lines represent the per capita gross domestic product (GDP) for San Francisco ($81,347 in 2015 [57]) and three times the per capita GDP, respectively, two thresholds denoting a very cost-effective and cost-effective use of resources, by international standards [56]. *PrEP* pre-exposure prophylaxis, *ART* antiretroviral therapy, *ICER* incremental cost-effectiveness ratio, *QALY* quality-adjusted life-year, *GDP* gross domestic product

Our findings are qualitatively robust to parameter uncertainty. In sensitivity analysis, we found that the PrEP costs and coverage had the largest impact on PrEP cost-effectiveness for the combination of high PrEP coverage and earlier ART compared to the status quo (Fig. 5). If the annual costs of PrEP were 50% lower than the base case, the ICER would decrease from $115,320 to $54,027 per QALY gained. Increasing PrEP coverage from the low to high scenario in the presence of earlier ART would increase the ICER from $4745 to $115,320 per QALY gained. However, the ICER is less sensitive to other parameters (Additional file 1: Supplementary Material).

## Discussion

We found that expanding PrEP while initiating ART earlier (at 1 year post-infection versus the status quo of 1.6 years post-infection) could provide substantial, cost-effective health benefits to the San Francisco MSM population. However, the most cost-effective intervention is earlier ART without further PrEP expansion. This would prevent 9700 (22%) total new infections and 1457 (8%) new drug-resistant infections, and add 43,649 QALYs over the next 20 years; this is very cost-effective at $4745 per QALY gained. Expanding PrEP coverage in addition to earlier ART would further reduce incidence, but only add 1.7–2.4 times more QALYs while costing 28–58 times more than earlier ART alone (Table 1). Still, high PrEP coverage plus earlier ART ($115,320 per QALY gained) is cost-effective for San Francisco even considering the acquisition and transmission of ART-mediated drug resistance. For a budget under $470 million annually, the best strategy is to keep current strategies of PrEP enrollment while initiating ART earlier. This may be advisable for San Francisco, based on the city’s 2015–2016 annual HIV/AIDS budget [58] (though this excludes private insurance and may therefore underestimate the total funding available). A higher budget would suggest that PrEP expansion in addition to earlier ART is advisable.

Our estimated ICER of expanding PrEP without earlier initiation of ART of $117,130–132,520 per QALY gained (Table 1) is far greater than estimates for targeted high-risk MSM interventions in New York City ($31,970 [20]), Los Angeles County ($27,863–37,181 [26]), and nationwide US ($52,443 [23]), but lower than estimates for the general MSM population in New York City ($353,739 [22]) and nationwide US ($172,091–216,480 [23]). These discrepancies may stem from the different settings, PrEP targeting strategies, or model assumptions. For example, San Francisco has higher testing and treatment rates than New York City and the entire US [10, 18, 22, 23]. We assume two-fold higher life expectancy gains following treatment than Juusola et al. [23], based on more recent life expectancy estimates [37, 38]. Recent reviews discussed variation amongst ICER calculations in greater detail [28, 59].

Drabo et al. [26] conducted a similar study for high-risk MSM in Los Angeles, but used a different model structure. Our infection–age-structured model allows us to track the life expectancy at different ART initiation times and among individuals with drug sensitive or resistant strains (the primary innovation of our model). In addition, their model initiated ART treatment at a CD4+ cell count ≤ 500 cells/mm^3^, whereas we considered an even earlier ART strategy. Finally, we accounted for the switch to second-line drug treatment after treatment failure and its associated costs. Despite these differences, we concur that all combinations of earlier ART and PrEP coverage are cost-effective to varying degrees, and that the optimal strategy depends on budgetary constraints.

Our study has several limitations. First, to explore the cost-effectiveness of the above scenarios, we fit our model to multiple sources of data, including incidence of AIDS diagnoses and deaths, HIV prevalence, the fraction of drug-resistance amongst incident cases, and other demographic data [18]. Fitting to so many sources data has the advantage of integrating much of what is known about this epidemic but is ambitious in the sense that it is difficult to closely fit all data sets simultaneously. One possibility to solving this would have been to fit only a subset of these data such that model fits would have better reflected those datasets but not been informed by others. Second, we did not model PrEP-mediated acquired drug resistance. Our results should be robust to this exclusion, as recommended screening practices are thought to limit PrEP-acquired resistance via quick identification of breakthrough infections amongst individuals on PrEP [21, 43, 60]. Third, we assumed that sexual partners were assorted independent of PrEP or ART usage. If PrEP were targeted towards individuals whose partners, if infected, were less likely to be on ART, then PrEP would be expected to be more effective and cost-effective. Should individuals on PrEP be more likely to engage in sexual partnerships with infected individuals on ART than with those not on ART, then this would reduce the estimated cost-effectiveness of PrEP. Fourth, we assumed a homogenous PrEP coverage for the entire population irrespective of risk and age. We made this assumption for simplicity and due to the lack of information about age- and risk-group mixing contact patterns. If PrEP coverage is higher in individuals within the MSM population who are at higher risk of infection than the average (i.e., because of PrEP targeting to high-risk groups or high-risk individuals seeking out PrEP), then PrEP would be more cost-effective. In contrast, if high-risk groups also constitute groups that are hard to reach with PrEP, then PrEP cost-effectiveness would be lower than estimated. Similarly, the indirect effects of PrEP on the population would depend on the propensity to transmit of individuals on PrEP, should they have been infected if they were not on PrEP. Finally, while our results were qualitatively robust to parameter uncertainty, future changes in intervention costs or efficacy may affect our estimates.

## Conclusions

In summary, expanding PrEP coverage and shifting ART initiation even earlier in San Francisco would reduce the total and drug resistant incidence, and add over 40,000 QALYs to the population over 20 years. Combining higher PrEP coverage and earlier ART is predicted to be cost-effective at three times the per capita GDP threshold. These results are robust across a wide range of assumptions regarding drug resistance and the effectiveness and cost of second-line drug regimens.

## Additional file

Additional file 1: Supplementary materials used to describe model details and parameters estimation. (PDF 400 kb)

## Abbreviations

ART: antiretroviral therapy
GDP: gross domestic product
ICER: incremental cost-effectiveness ratio
MSM: men who have sex with men
PrEP: pre-exposure prophylaxis
QALY: quality-adjusted life-year

## References

[1] Holmes D (2012). FDA paves the way for pre-exposure HIV prophylaxis. Lancet.

[2] Abdool Karim Q, Abdool Karim SS, Frohlich JA (2010). Effectiveness and safety of tenofovir gel, an antiretroviral microbicide, for the prevention of HIV infection in women. Science.

[3] Grant RM, Lama JR, Anderson PL (2010). Preexposure chemoprophylaxis for HIV prevention in men who have sex with men. N Engl J Med.

[4] Molina JM, Capitant C, Spire B (2015). On-demand preexposure prophylaxis in men at high risk for HIV-1 infection. N Engl J Med.

[5] Grant RM, Anderson PL, McMahan V (2014). Uptake of pre-exposure prophylaxis, sexual practices, and HIV incidence in men and transgender women who have sex with men: a cohort study. Lancet Infect Dis.

[6] McCormack S, Dunn DT, Desai M (2016). Pre-exposure prophylaxis to prevent the acquisition of HIV-1 infection (PROUD): effectiveness results from the pilot phase of a pragmatic open-label randomised trial. Lancet.

[7] Grant RM, Liu A, Hecht J, et al. Scale-up of pre-exposure prophylaxis in San Francisco to impact HIV incidence. In: Conference on Retroviruses and Opportunistic Infections, Seattle, Washington. 2015. http://www.croiconference.org/sessions/scale-preexposure-prophylaxis-san-francisco-impact-hiv-incidence.

[8] Chen YH, Snowden JM, McFarland W, Raymond HF. Pre-exposure prophylaxis (PrEP) use, seroadaptation, and sexual behavior among men who have sex with men, San Francisco, 2004-2014. AIDS Behav. 2016;20:2791–7. 10.1007/s10461-016-1357-2 26983951

[9] Highleyman L. At least 6000 people thought to be on HIV PrEP in San Francisco. http://hivandhepatitis.com/hiv-prevention/hiv-prep/5730-at-least-6000-people-thought-to-be-on-hiv-prep-in-san-francisco.

[10] San Francisco Department of Public Health Population Health Division HIV Epidemiology Section. HIV Epidemiology Annual Report. 2015. https://www.sfdph.org/dph/files/reports/RptsHIVAIDS/AnnualReport2015-20160831.pdf. Accessed 5 Nov 2016.

[11] Grey JA, Bernstein KT, Sullivan PS (2016). Estimating the population sizes of men who have sex with men in US states and counties using data from the American community survey. JMIR Public Health Surveill.

[12] San Francisco Department of Public Health HIV Epidemiology Section (2010). HIV/AIDS Epidemiology Annual Report.

[13] Cohen MS, Chen YQ, McCauley M (2011). Prevention of HIV-1 infection with early antiretroviral therapy. N Engl J Med.

[14] Supervie V, García-Lerma JG, Heneine W, Blower S (2010). HIV, transmitted drug resistance, and the paradox of preexposure prophylaxis. Proc Natl Acad Sci U S A.

[15] Supervie V, Barrett M, Kahn JS (2011). Modeling dynamic interactions between pre-exposure prophylaxis interventions & treatment programs: predicting HIV transmission & resistance. Sci Rep.

[16] Abbas UL, Anderson RM, Mellors JW (2007). Potential impact of antiretroviral chemoprophylaxis on HIV-1 transmission in resource-limited settings. PLoS One.

[17] Shen MW, Xiao YN, Rong LB (2015). Global stability of an infection-age structured HIV-1 model linking within-host and between-host dynamics. Math Biosci.

[18] Shen MW, Xiao YN, Rong LB, Meyers LA, Bellan SE (2017). Early antiretroviral therapy and potent second-line drugs could decrease HIV incidence of drug resistance. P Roy Soc B-Biol Sci.

[19] Adamson BJ, Bounthavong M, Kublin JG, Garrison L (2015). Cost-effectiveness analysis of a partially effective HIV vaccine in San Francisco. Value Health.

[20] Desai K, Sansom SL, Ackers ML (2008). Modeling the impact of HIV chemoprophylaxis strategies among men who have sex with men in the United States: HIV infections prevented and costeffectiveness. AIDS.

[21] Paltiel AD, Freedberg KA, Scott CA (2009). HIV preexposure prophylaxis in the United States: impact on lifetime infection risk, clinical outcomes, and cost-effectiveness. Clin Infect Dis.

[22] Koppenhaver RT, Sorensen SW (2011). The cost-effectiveness of pre-exposure prophylaxis in men who have sex with men in the United States: an epidemic model. J Acquir Immune Defic Syndr.

[23] Juusola JL, Brandeau ML, Owens DK, Bendavid E (2012). The cost-effectiveness of preexposure prophylaxis for HIV prevention in the United States in men who have sex with men. Ann Intern Med.

[24] Kessler J, Myers JE, Nucifora KA (2014). Averting HIV infections in New York City: a modeling approach estimating the future impact of additional behavioral and biomedical prevention strategies. AIDS.

[25] Ross EL, Cinti SK, Hutton DW (2016). Implementation and operational research: a cost-effective, clinically actionable strategy for targeting HIV preexposure prophylaxis to high-risk men who have sex with men. J Acquir Immune Defic Syndr.

[26] Drabo EF, Hay JW, Vardavas R, Wagner ZR, Sood N. A cost-effectiveness analysis of preexposure prophylaxis for the prevention of HIV among Los Angeles County men who have sex with men. Clin Infect Dis. 2016;63:1495–504. 10.1093/cid/ciw578 27558571

[27] Bernard CL, Brandeau ML, Humphreys K (2016). Cost-effectiveness of HIV preexposure prophylaxis for people who inject drugs in the United States. Ann Intern Med.

[28] Gomez GB, Borquez A, Case KK, Wheelock A, Vassall A, Hankins C (2013). The cost and impact of scaling up pre-exposure prophylaxis for HIV prevention: a systematic review of cost-effectiveness modelling studies. PLoS Med.

[29] Anderson SJ, Cherutich P, Kilonzo N (2014). Maximising the effect of combination HIV prevention through prioritisation of the people and places in greatest need: a modelling study. Lancet.

[30] Nichols BE, Baltussen R, van Dijk JH (2014). Cost-effectiveness of PrEP in HIV/AIDS control in Zambia: a stochastic league approach. J Acquir Immune Defic Syndr.

[31] Hallett TB, Baeten JM, Heffron R (2011). Optimal uses of antiretrovirals for prevention in HIV-1 serodiscordant heterosexual couples in South Africa: a modelling study. PLoS Med.

[32] Kim SB, Yoon M, Ku NS (2014). Mathematical modeling of HIV prevention measures including pre-exposure prophylaxis on HIV incidence in South Korea. PLoS One.

[33] Cremin I, Alsallaq R, Dybul M, Piot P, Garnett G, Hallett TB (2013). The new role of antiretrovirals in combination HIV prevention: a mathematical modelling analysis. AIDS.

[34] Smith JA, Anderson SJ, Harris KL (2016). Maximising HIV prevention by balancing the opportunities of today with the promises of tomorrow: a modelling study. Lancet HIV.

[35] Ramadhani HO, Bartlett JA, Thielman NM (2014). Association of first-line and second-line antiretroviral therapy adherence. Open Forum Infect Dis.

[36] Dodd PJ, Garnett GP, Hallett TB (2010). Examining the promise of HIV elimination by ‘test and treat’ in hyperendmic settings. AIDS.

[37] May M, Gompels M, Sabin C (2012). Life expectancy of HIV-1-positive individuals approaches normal, conditional on response to antiretroviral therapy: UK collaborative HIV cohort study. J Int AIDS Soc.

[38] May M, Gompels M, Delpech V (2014). Impact on life expectancy of HIV-1 positive individuals of CD4+ cell count and viral load response to antiretroviral therapy. AIDS.

[39] Richman DD, Morton SC, Wrin T (2004). The prevalence of antiretroviral drug resistance in the United States. AIDS.

[40] Jiang J, Yang X, Ye L (2014). Pre-exposure prophylaxis for the prevention of HIV infection in high risk populations: a meta-analysis of randomized controlled trials. PLoS One.

[41] Liu A, Cohen S, Follansbee S (2014). Early experiences implementing preexposure prophylaxis (PrEP) for HIV prevention in San Francisco. PLoS Med.

[42] Lehman DA, Baeten JM, McCoy CO (2015). Risk of drug resistance among persons acquiring HIV within a randomized clinical trial of single- or dual-agent preexposure prophylaxis. J Infect Dis.

[43] Grant RM, Liegler T, Defechereux P (2015). Drug resistance and plasma viral RNA level after ineffective use of oral pre-exposure prophylaxis in women. AIDS.

[44] van de Vijver DA, Nichols BE, Abbas UL (2013). Preexposure prophylaxis will have a limited impact on HIV-1 drug resistance in sub-Saharan Africa: a comparison of mathematical models. AIDS.

[45] Abbas UL, Glaubius R, Mubayi A, Hood G, Mellors JW (2013). Antiretroviral therapy and preexposure prophylaxis: combined impact on HIV-1 transmission and drug resistance in South Africa. J Infect Dis.

[46] Weis JF, Baeten JM, McCoy CO (2016). Preexposure prophylaxis-selected drug resistance decays rapidly after drug cessation. AIDS.

[47] Zetola NM, Bernstein K, Ahrens K (2009). Using surveillance data to monitor entry into care of newly diagnosed HIV-infected persons: San Francisco, 2006-2007. BMC Public Health.

[48] Tengs TO, Lin TH (2002). A meta-analysis of utility estimates for HIV/AIDS. Med Decis Mak.

[49] Long EF, Brandeau ML, Owens DK (2009). Potential population health outcomes and expenditures of HIV vaccination strategies in the United States. Vaccine.

[50] Long EF, Brandeau ML, Owens DK (2010). The cost-effectiveness and population outcomes of expanded HIV screening and antiretroviral treatment in the United States. Ann Intern Med.

[51] Juusola JL, Brandeau ML, Long EF, Owens DK, Bendavid E (2011). The costeffectiveness of symptom-based testing and routine screening for acute HIV infection in men who have sex with men in the USA. AIDS.

[52] Nichols BE, Sigaloff KC, Kityo C (2014). Increasing the use of second-line therapy is a cost-effective approach to prevent the spread of drug-resistant HIV: a mathematical modelling study. J Int AIDS Soc.

[53] Solem CT, Snedecor SJ, Khachatryan A (2014). Cost of treatment in a US commercially insured, HIV-1-infected population. PLoS One.

[54] Horberg M, Raymond B (2013). Financial policy issues for HIV pre-exposure prophylaxis: cost and access to insurance. Am J Prev Med.

[55] Drummond M, Sculpher M, Torrance G, O'Brien B, Stoddart G (2005). Methods for the economic evaluation of health care programmes.

[56] World Health Organization. The World Health Report 2002. Reducing Risks, Promoting Healthy Life. http://www.who.int/whr/2002/en. Accessed 5 Nov 2016.

[57] Bureau of Economic Analysis US Department of Commerce. Per capita real GDP by metropolitan area. San Francisco-Oakland-Hayward, CA (Metropolitan Statistical Area). http://www.bea.gov/iTable/iTable.cfm?reqid=70&step=1&isuri=1&acrdn=2#reqid=70&step=10&isuri=1&7003=1000&7035=1&7004=naics&7005=1&7006=41860&7036=-1&7001=21000&7002=2&7090=70&7007=2015&7093=levels. Accessed 5 Nov 2016.

[58] Lee EM. Mayor Lee Announces HIV/AIDS Funding in City's Budget. http://sfmayor.org/article/mayor-lee-announces-hivaids-funding-citys-budget. Accessed 5 Nov 2016.

[59] Schackman BR (2012). Cost-effectiveness of pre-exposure prophylaxis for HIV: a review. Curr Opin HIV AIDS.

[60] Centers for Disease Control and Prevention, U.S. Public Health Service (2014). Preexposure prophylaxis for the prevention of HIV infection in the United States-2014: A Clinical Practice Guideline.

