# Supplementary material for: The cost-effectiveness of oral HIV pre-exposure prophylaxis and early antiretroviral therapy in the presence of drug resistance among men who have sex with men in San Francisco
Source: BMC Med. 2018 Apr 24;16:58. doi: 10.1186/s12916-018-1047-1 (PMC5914040; doi:10.1186/s12916-018-1047-1)
Supplement: Supplementary file 1 — Supplementary materials used to describe model details and parameters estimation. (PDF 400 kb) [file 12916_2018_1047_MOESM1_ESM.pdf]

Supplementary Appendix: The Cost-Effectiveness of Oral HIV Pre-Exposure Prophylaxis and Early Antiretroviral Therapy in the Presence of Drug Resistance Among Men Who Have Sex With Men in San Francisco

Mingwang Shen[1,2,3], Yanni Xiao[2*], Libin Rong[4], Lauren Ancel Meyers[3,5], Steven E. Bellan[6,7]

1 School of Public Health, Xi'an Jiaotong University Health Science Center, Xi'an, Shaanxi, 710061, PR China

2 School of Mathematics and Statistics, Xi'an Jiaotong University, Xi'an, Shaanxi, 710049, PR China

3 Department of Integrative Biology, The University of Texas at Austin, Austin, Texas 78712, USA

4 Department of Mathematics, University of Florida, Gainesville, FL 32611, USA

5 The Santa Fe Institute, Santa Fe, New Mexico 87501, USA

6 Department of Epidemiology and Biostatistics, College of Public Health, University of Georgia, Athens, Georgia 30602, USA

7 Center for Ecology of Infectious Diseases, University of Georgia, Athens, Georgia 30602, USA

This is a supplementary document describing mathematical details and analytical derivations used for our results presented in the main text and parameter estimation. In section 1, we present our mathematical model which describes the dynamics of PrEP use in MSM population when considering the transmission and acquisition of drug resistance. It is followed by sections of health and economic outcomes. How the parameters are chosen or estimated is given in section 4. In section 5, we provide more tables and figures to support the results in the main text.

Contents

1 Model formulation **2**

2 Health outcomes: New infections, prevalence and the fraction of new infections that are drug resistant **5**

2.1 New infections 5

2.2 Prevalence 6

2.3 The fraction of new infections that are drug resistant 6

3 Economic outcomes: QALYs, Costs, and ICERs **6**

3.1 QALYs 6

3.2 Costs 6

3.3 ICERs 7

4 Parameter estimation **8**

5 Supplementary Tables and Figures **10**

5.1 Supplementary Tables 10

5.2 Supplementary Figures 16

*Corresponding author. E-mail: yxiao@mail.xjtu.edu.cn, Tel:+86 29 82663156, Fax:+86 29 82668551

1 Model formulation

We extended our previously developed model [1, 2] by considering the use of PrEP among male homosexual population in San Francisco which is decomposed into twelve categories (see **Figure 1(a)** in the main text and **Appendix Figure 1**): susceptible individuals without PrEP (S) and with PrEP (P), untreated individuals infected with drug-sensitive strains at the primary stage (I_{s1}^U), chronic stage (I_{s2}^U) and AIDS stage (I_{s3}^U), untreated drug-resistant cases at the primary stage (I_{r1}^U), chronic stage (I_{r2}^U) and AIDS stage (I_{r3}^U), individuals treated with combination antiretroviral therapy (ART) but did not develop drug resistance (I_{s1}^T) and those who have entered the AIDS stage (I_{s2}^T), and ART-treated individuals with drug resistance before the AIDS stage (I_{r1}^T) and at the AIDS stage (I_{r2}^T). The variable's subscript identifies whether the infection is drug-sensitive (s) or drug-resistant (r); the superscript specifies whether the individuals are treated with ART (T) or untreated (U).

Denote the duration of the primary, chronic, AIDS stage for untreated drug-sensitive individuals as a_p, d_c, d_A, and assume untreated drug-resistant individuals have a longer chronic stage $d_r(\geq d_c)$ due to weaker viral replication capacity (lower viral load in the absence of drug pressure) and thus a longer life expectancy [3, 4]. We assume that the duration of the primary and AIDS stages did not differ with or without treatment and resistance, as in [1,5]. Let $A_s^U(= a_p + d_c), A_r^U(= a_p + d_r), A_s^T, A_r^T$ and $D_s^U(= A_s^U + d_A), D_r^U(= A_r^U + d_A), D_s^T, D_r^T$ be the time when the AIDS stage starts and the infected individual dies because of AIDS for untreated drug-sensitive, untreated drug-resistant, treated drug-sensitive and treated drug-resistant individuals, respectively. Assume that treatment starts at time a_{ART} after infection irrespective of being infected with sensitive or resistant strains. Uninfected individuals are recruited into the susceptible population at a positive constant rate b. People exit the sexually-active population at a positive constant rate m due to behavior changes. The infected individuals at the chronic stage are assumed to receive antiviral treatment with a rate $\eta = 1/(a_{ART} - a_p)$ (For example, if all infected individuals are treated at an annual rate of 50%, then the average interval between infection and ART initiation is 2 years [6]). The parameter f_r is the fraction of treated individuals who develop drug resistance and we assume that all of these drug-resistant cases use second-line drugs. Let t denote time and a denote the infection age. We assume that all of the infected individuals with the same infection age are homogeneous and have the same rates.

Let $i_{qj}^U(a,t)$, $i_{q1}^T(a,t)$ and $i_{q2}^T(a,t)$ (where $j = 1, 2, 3$ and $q \in \{s, r\}$) be the respective density of infected individuals in I_{qj}^U, I_{q1}^T and I_{q2}^T classes at time t and infection age a. It follows that

$$\begin{aligned} I_{q1}^U(t) &= \int_0^{a_p} i_{q1}^U(a,t)da, I_{q2}^U(t) = \int_{a_p}^{A_q^U} i_{q2}^U(a,t)da, I_{q3}^U(t) = \int_{A_q^U}^{D_q^U} i_{q3}^U(a,t)da, \\ I_{q1}^T(t) &= \int_{a_{ART}}^{A_q^T} i_{q1}^T(a,t)da, I_{q2}^T(t) = \int_{A_q^T}^{D_q^T} i_{q2}^T(a,t)da, \ q \in \{s, r\}, \end{aligned} \tag{1}$$

are the number of infected individuals in I_{qj}^U, I_{q1}^T and I_{q2}^T classes ($j = 1, 2, 3$ and $q \in \{s, r\}$), respectively, at time $t \geq 0$. We denote the disease-induced mortality rates in the classes $I_{s2}^U, I_{r2}^U, I_{s1}^T, I_{r1}^T$ and AIDS classes (including $I_{s3}^U, I_{r3}^U, I_{s2}^T, I_{r2}^T$) as $\mu_s^U, \mu_r^U, \mu_s^T, \mu_r^T$ and μ_A, respectively.

The probability that an infected individual in the I_{qj}^U, I_{q1}^T and I_{q2}^T class (where $j = 1, 2, 3$ and $q \in \{s, r\}$) still

stays in the original class at infection age a [1] is given by

$$
\begin{aligned}
&\sigma_{q1}^{U}(a) = e^{-\int_0^a (m+\delta_1)ds}, \ a \in [0, a_p], \ q \in \{s,r\},\\
&\sigma_{q2}^{U}(a) = e^{-\int_{a_p}^a (m+\mu_q^U+\delta_q^U+\eta)ds}, \ a \in [a_p, A_q^U], \ q \in \{s,r\},\\
&\sigma_{q3}^{U}(a) = e^{-\int_{A_q^U}^a (m+\mu_A)ds}, \ a \in [A_q^U, D_q^U], \ q \in \{s,r\},\\
&\sigma_{q1}^{T}(a) = e^{-\int_{a_{ART}}^a (m+\mu_q^T+\delta_q^T)ds}, \ a \in [a_{ART}, A_q^T], \ q \in \{s,r\},\\
&\sigma_{q2}^{T}(a) = e^{-\int_{A_q^T}^a (m+\mu_A)ds}, \ a \in [A_q^T, D_q^T], \ q \in \{s,r\},
\end{aligned} \tag{2}
$$

where δ_1 is the progression rate to the chronic stage and δ_q^U, δ_q^T ($q \in \{s,r\}$) are the progression rates to the AIDS stage for untreated and treated individuals, respectively.

Let F_q be the fraction of the untreated population that receives treatment [3]. It is given by

$$F_q = \frac{\eta}{m + \mu_q^U + \delta_q^U + \eta}, q \in \{s,r\}. \tag{3}$$

Denote the transmission rate at the primary stage, chronic stage and AIDS stage for untreated drug-sensitive and drug-resistant individuals as $\beta_s^p, \beta_s^U, \beta_s^A$, and $\beta_r^p, \beta_r^U, \beta_r^A$, respectively. The transmission rate of a treated drug-sensitive (β_s^T) or drug-resistant (β_r^T) case is the infectivity of an untreated individual (β) multiplied by a constant, i.e., $\beta_s^T = \alpha_s \beta_s^U$ and $\beta_r^T = \alpha_r \beta_s^U$ where $\alpha_s \leq \alpha_r$, i.e., the second-line drug effectiveness $1 - \alpha_r$ is assumed to be lower than the first-line drug effectiveness $1 - \alpha_s$ (see Appendix Figures 4-5 in [2]) due to lower adherence [7].

Patients starting ART with higher baseline CD4 counts had longer life expectancies [5,8–11]. The relationship between prior-treatment CD4+ count and infection age shown in Fig. 1(B) in [12] also suggested that a higher CD4+ count corresponded to an earlier infection stage. Thus, the earlier ART starts, the longer the patient is expected to live and vice versa. Similar to the assumption in [5], we assume that the duration from treatment initiation to death for treated individuals is a linear decreasing function of ART initiation timing a_{ART} as follows (see Appendix Figure 3 in [2]):

$$L_q^T = L_q^0 - \xi_q^T a_{ART}, \ q \in \{s,r\}, \tag{4}$$

where L_q^0 is the average maximum expectancies for those who are treated immediately after infection (i.e.,$a_{ART} = 0$) and ξ_q^T is the slope. Therefore, we have

$$D_q^T = a_{ART} + L_q^T, A_q^T = D_q^T - d_A, \ q \in \{s,r\}. \tag{5}$$

We assumed the PrEP effectiveness against drug-sensitive strains (defined as the reduction in susceptibility to HIV infection upon exposure to an HIV-infected drug-sensitive partner) was ϵ_s. We defined PrEP effectiveness to represent the product of PrEP's biomedical efficacy and PrEP adherence. We assumed PrEP effectiveness against resistant strains was $\epsilon_r = \delta_r \epsilon_s$ (δ_r is defined as relative effectiveness of PrEP, i.e., the ratio of PrEP effectiveness against drug-resistant strains ϵ_r relative to drug-sensitive strains ϵ_s) [13–15].

We develop the complete dynamical model as follows

$$
\begin{cases}
\dfrac{dS(t)}{dt} = b - mS(t) - \dfrac{S(t)}{N(t)} \displaystyle\sum_{q\in\{s,r\}} \left(\int_0^{a_p} \beta_q^p i_{q1}^U(a,t)da + \int_{a_p}^{A_q^U} \beta_q^U i_{q2}^U(a,t)da + \int_{A_q^U}^{D_q^U} \beta_q^A i_{q3}^U(a,t)da \right. \\
\qquad \left. + \displaystyle\int_{a_{ART}}^{A_q^T} \beta_q^T i_{q1}^T(a,t)da + \int_{A_q^T}^{D_q^T} \beta_q^A i_{q2}^T(a,t)da \right), \\
\dfrac{dP(t)}{dt} = \xi S(t) - wP(t) - mP(t) - \dfrac{P(t)}{N(t)} \displaystyle\sum_{q\in\{s,r\}} (1-\epsilon_q) \left[\int_0^{a_p} \beta_q^p i_{q1}^U(a,t)da + \int_{a_p}^{A_q^U} \beta_q^U i_{q2}^U(a,t)da \right. \\
\qquad \left. + \displaystyle\int_{A_q^U}^{D_q^U} \beta_q^A i_{q3}^U(a,t)da + \int_{a_{ART}}^{A_q^T} \beta_q^T i_{q1}^T(a,t)da + \int_{A_q^T}^{D_q^T} \beta_q^A i_{q2}^T(a,t)da \right], \\
\dfrac{\partial i_{q1}^U(a,t)}{\partial t} + \dfrac{\partial i_{q1}^U(a,t)}{\partial a} = -(m+\delta_1) i_{q1}^U(a,t), \quad 0 < a \le a_p,\ q \in \{s,r\}, \\
\dfrac{\partial i_{q2}^U(a,t)}{\partial t} + \dfrac{\partial i_{q2}^U(a,t)}{\partial a} = -(m+\mu_q^U+\delta_q^U+\eta) i_{q2}^U(a,t), \quad a_p < a \le A_q^U,\ q \in \{s,r\}, \\
\dfrac{\partial i_{q3}^U(a,t)}{\partial t} + \dfrac{\partial i_{q3}^U(a,t)}{\partial a} = -(m+\mu_A) i_{q3}^U(a,t), \quad A_q^U < a \le D_q^U,\ q \in \{s,r\}, \\
\dfrac{\partial i_{q1}^T(a,t)}{\partial t} + \dfrac{\partial i_{q1}^T(a,t)}{\partial a} = -(m+\mu_q^T+\delta_q^T) i_{q1}^T(a,t),\ a_{ART} < a \le A_q^T,\ q \in \{s,r\}, \\
\dfrac{\partial i_{q2}^T(a,t)}{\partial t} + \dfrac{\partial i_{q2}^T(a,t)}{\partial a} = -(m+\mu_A) i_{q2}^T(a,t),\ A_q^T < a \le D_q^T,\ q \in \{s,r\}, \\
i_{q1}^U(0,t) = \dfrac{S(t)+(1-\epsilon_q)P(t)}{N(t)} \left(\displaystyle\int_0^{a_p} \beta_q^p i_{q1}^U(a,t)da + \int_{a_p}^{A_q^U} \beta_q^U i_{q2}^U(a,t)da + \int_{A_q^U}^{D_q^U} \beta_q^A i_{q3}^U(a,t)da \right. \\
\qquad \left. + \displaystyle\int_{a_{ART}}^{A_q^T} \beta_q^T i_{q1}^T(a,t)da + \int_{A_q^T}^{D_q^T} \beta_q^A i_{q2}^T(a,t)da \right),\ q \in \{s,r\}, \\
i_{q2}^U(a_p,t) = \displaystyle\int_0^{a_p} \delta_1 i_{q1}^U(a,t)da,\ q \in \{s,r\}, \\
i_{q3}^U(A_q^U,t) = \displaystyle\int_{a_p}^{A_q^U} \delta_q^U i_{q2}^U(a,t)da,\ q \in \{s,r\}, \\
i_{s1}^T(a_{ART},t) = (1-f_r) \displaystyle\int_{a_p}^{A_s^U} \eta i_{s2}^U(a,t)da, \\
i_{r1}^T(a_{ART},t) = f_r \displaystyle\int_{a_p}^{A_s^U} \eta i_{s2}^U(a,t)da + \int_{a_p}^{A_r^U} \eta i_{r2}^U(a,t)da, \\
i_{q2}^T(A_q^T,t) = \displaystyle\int_{a_{ART}}^{A_q^T} \delta_q^T i_{q1}^T(a,t)da,\ q \in \{s,r\}, \\
S(0) = S_0 \ge 0, i_{qj}^U(a,0) = i_{qj0}^U(a), j=1,2,3, q \in \{s,r\}; i_{q1}^T(a,0) = i_{q10}^T(a), i_{q2}^T(a,0) = i_{q20}^T(a), q \in \{s,r\}.
\end{cases} \tag{6}
$$

where $i_{q10}^U(a) \in L_+^1(0,a_p), i_{q20}^U(a) \in L_+^1(a_p, A_q^U), i_{q30}^U(a) \in L_+^1(A_q^U, D_q^U), i_{q10}^T(a) \in L_+^1(a_{ART}, A_q^T), i_{q20}^T(a) \in L_+^1(A_q^T, D_q^T), q \in \{s,r\}$. Here L_+^1 is the space of functions that are nonnegative and Lebesgue integrable over the specified interval.

The number of persons living with HIV/AIDS at any time t is given by

$$
I_{total}(t) = \sum_{q\in\{s,r\}} \left(\sum_{j=1}^{3} I_{qj}^U(t) + I_{q1}^T(t) + I_{q2}^T(t) \right). \tag{7}
$$

The total population size at any time t is given by

$$
N(t) = S(t) + P(t) + I_{total}(t). \tag{8}
$$

The PrEP coverage among susceptible MSM population among any time t is given by

$$\text{PrEP coverage}(t) = \frac{P(t)}{S(t)+P(t)}. \tag{9}$$

Notice that we keep track of the numbers of newly diagnosed AIDS cases and AIDS deaths at time t using the following equations:

$$\begin{aligned} Cases(t) &= \sum_{q\in\{s,r\}} \left(\int_{a_p}^{A_q^U} \delta_q^U i_{q2}^U(a,t)da + \int_{a_{ART}}^{A_q^T} \delta_q^T i_{q1}^T(a,t)da \right), \\ Deaths(t) &= \sum_{q\in\{s,r\}} \left(\int_{A_q^U}^{D_q^U} \mu_A i_{q3}^U(a,t)da + \int_{A_q^T}^{D_q^T} \mu_A i_{q2}^T(a,t)da \right). \end{aligned} \tag{10}$$

There are two ways to model the development of drug resistance. The first way is to model the progression of untreated $\longrightarrow$ treated sensitive $\longrightarrow$ treated resistant. The second is to model the consequence of treatment, assuming that if resistance is acquired then it happens fast enough such that amongst those who develop resistance, it is not important to consider that they move through a treated sensitive state. In this formulation, a fraction of treated patients will develop drug resistance after ART treatment and a fraction won't. In this paper, we adopted the second choice. We did this because it allows us to assume that a fraction (f_r) of treated individuals will acquire drug resistance, as shown in **Figure 1(a)** in the main text and **Appendix Figure 1**. With the first method, drug resistance is developed at a certain rate, and the proportion whoever develop drug resistance would also depend on other rates of the model, making it more difficult to match the model to available data.

A second advantage of using the second method is that we can track the timing of ART administration easily for both treated drug-sensitive and drug-resistant individuals using our infection-age-structured model. We used the same modeling approach in our earlier work [2] and adapt the parameter estimates from that paper here. If we adopt first approach (a rate of acquiring drug resistance post-treatment), it would be hard to determine the time at which drug-resistant people initiate treatment because, while the time at which drug-sensitive people develop drug resistance would vary substantially, there would be no way to track this in the model. It also follows from the sensitivity analysis (**Figure 5** in the main text and **Appendix Figures 4-7**) that the fraction (f_r) of acquired drug resistance has a very minor effect on ICER. Thus, we believe either modeling approach would lead to similar cost-effectiveness estimates though the one we chose makes other assumptions of our model easier to execute.

2 Health outcomes: New infections, prevalence and the fraction of new infections that are drug resistant

2.1 New infections

The (undiscounted) cumulative number of total new HIV infections that occur in the entire population over the time horizon T ($T = 20$ years in this paper, i.e., from 2018 to 2038) is calculated by

$$\text{Total new infecions over } T \text{ years} = \int_0^T (i_{s1}^U(0,t) + i_{r1}^U(0,t))dt, \tag{11}$$

and the cumulative number of new drug-resistant infections over T years is calculated by

$$\text{New drug-resistant infecions over } T \text{ years} = \int_0^T i_{r1}^U(0,t)dt, \tag{12}$$

where $i_{q1}^U(0,t), q \in \{s,r\}$ is the new infections at time t shown in Eq. (6).

2.2 Prevalence

The HIV prevalence at any time t is given by

$$Prevalence(t) = \frac{I_{total}(t)}{N(t)}, \tag{13}$$

where $I_{total}(t)$ and $N(t)$ are given by Eqs. (7) and (8) respectively.

2.3 The fraction of new infections that are drug resistant

The prevalence of transmitted drug resistance (TDR) among newly infected individuals (the fraction of new infections that are drug resistant) at any time t is given by

$$TDR(t) = \frac{i_{r1}^U(0,t)}{i_{s1}^U(0,t) + i_{r1}^U(0,t)}, \tag{14}$$

where $i_{q1}^U(0,t), q \in \{s,r\}$ is the new infections at time t shown in Eq. (6).

3 Economic outcomes: QALYs, Costs, and ICERs

3.1 QALYs

Denote the quality of life of classes $S, P, I_{qj}^U, I_{q1}^T, I_{q2}^T, q \in \{s,r\}; j = 1,2,3$ as $Q_S, Q_P, Q_{qj}^U, Q_{q1}^T, Q_{q2}^T$, and the life discount rate as $\bar{r}$ (see **Appendix Table 1**), then we have the total health benefits for the entire population measured in discounted quality-adjusted life years (QALYs) during the intervention duration T (see [16,17]):

$$QALYs = \int_0^T e^{-\bar{r}t}\left(Q_S S(t) + Q_P P(t) + \sum_{q\in\{s,r\}}\left(\sum_{j=1}^{3} Q_{qj}^U I_{qj}^U(t) + Q_{q1}^T I_{q1}^T(t) + Q_{q2}^T I_{q2}^T(t)\right)\right)dt, \tag{15}$$

where $I_{qj}^U(t), I_{q1}^T(t), I_{q2}^T(t), q \in \{s,r\}; j = 1,2,3$ are given by Eq. (1). Here we assume that quality of life for drug-resistant individuals decreases by 5% relative to drug-sensitive individuals at the same stage [18,19], i.e., $Q_{rj}^U = 0.95Q_{sj}^U; j = 1,2,3$, $Q_{r1}^T = 0.95Q_{s1}^T$, and $Q_{r2}^T = 0.95Q_{s2}^T$.

3.2 Costs

Denote annual costs of PrEP as c_P, and annual HIV-related health care costs (exclude ART costs) for classes $I_{qj}^U, I_{q1}^T, I_{q2}^T, q \in \{s,r\}; j = 1,2,3$ as $c_{qj}^U, c_{q1}^T, c_{q2}^T, q \in \{s,r\}; j = 1,2,3$. Here, we assume the HIV-related health care costs for drug-sensitive individuals are the same as these for drug-resistant individuals, i.e., $c_{sj}^U = c_{rj}^U; j = 1,2,3$, $c_{s1}^T = c_{r1}^T$, and $c_{s2}^T = c_{r2}^T$. Let annual costs of ART (first-line drugs) for treated drug-sensitive individuals be c_s^{ART} and annual costs of second-line drugs for treated drug-resistant cases be c_r^{ART} (here we assume all drug-resistant individuals use second-line drugs). Assume ART was immediately following diagnosis so that the

test rate ϕ can be obtained as $\phi = 1/a_{ART}$. For uninfected individuals, the total costs of testing (antibody test) and counseling are denoted as c_{neg}. For infected individuals, the total costs of testing (antibody test) and counseling are denoted as c_{pos}, and the costs of diagnosis is denoted as c_{diag}. Denote the costs of genotype resistance test as c_{GT}. See **Appendix Table 1** for these costs. The cost discount rate is assumed as r. Then we calculate the total discounted costs for the entire population as the sum of annual health care costs for all individuals over the intervention's duration T as follows (see [16, 17]):

$$Costs = C_{HC} + C_{PrEP} + C_{ART}^{First\ line} + C_{ART}^{Second\ line} + C_{TC} + C_{Diag} + C_{GT}, \tag{16}$$

where

$$\begin{aligned}
C_{HC} &= \int_0^T e^{-rt} \sum_{q\in\{s,r\}} \left(\sum_{j=1}^{3} c_{qj}^U I_{qj}^U(t) + c_{q1}^T I_{q1}^T(t) + c_{q2}^T I_{q2}^T(t) \right) dt,\\
C_{PrEP} &= \int_0^T e^{-rt} c_P P(t) dt,\\
C_{ART}^{First\ line} &= \int_0^T e^{-rt} c_s^{ART} (I_{s1}^T(t) + I_{s2}^T(t)) dt,\\
C_{ART}^{Second\ line} &= \int_0^T e^{-rt} c_r^{ART} (I_{r1}^T(t) + I_{r2}^T(t)) dt,\\
C_{TC} &= \int_0^T e^{-rt} \left(c_{neg}\phi S(t) + c_{pos}\phi \sum_{q\in\{s,r\}} \sum_{j=1}^{3} I_{qj}^U(t) \right) dt,\\
C_{Diag} &= \int_0^T e^{-rt} c_{diag}\phi \sum_{q\in\{s,r\}} \sum_{j=1}^{3} I_{qj}^U(t) dt,\\
C_{GT} &= \int_0^T e^{-rt} c_{GT} \sum_{q\in\{s,r\}} \left(\sum_{j=1}^{3} \phi I_{qj}^U(t) + I_{q1}^T(t) + I_{q2}^T(t) \right) dt,
\end{aligned} \tag{17}$$

denote the total costs over T years respectively about (1) the HIV-related health care cost (C_{HC}), (2) the cost of PrEP (C_{PrEP}), (3) the cost of first-line ART ($C_{ART}^{First\ line}$), (4) the cost of second-line ART ($C_{ART}^{Second\ line}$), (5) the cost of testing, counseling (C_{TC}), (6) the cost of diagnosis (C_{Diag}), and (7) the cost of genotype resistance test (C_{GT}). See **Appendix Table 2** for details.

3.3 ICERs

We calculated the incremental cost-effectiveness ratio (ICER) of each intervention strategy, relative to the status quo as follows (see [16, 17, 20, 21]):

$$ICER = \frac{Costs_{Intervention} - Costs_{Status\ quo}}{QALYs_{Intervention} - QALYs_{Status\ quo}}. \tag{18}$$

In some cases, we also calculated the ICER of one intervention strategy A relative to another strategy B (for example, combination of high PrEP and earlier ART versus high PrEP alone).

$$ICER = \frac{Costs_A - Costs_B}{QALYs_A - QALYs_B}. \tag{19}$$

According to the WHO standards [22–24], if ICER<1 per capita GDP, the strategy is regarded as very cost-effective; if 1 per capita GDP<ICER<3 per capita GDP, the strategy is regarded as cost-effective; if ICER>3 per capita GDP, the strategy is regarded as not cost-effective.

4 Parameter estimation

We obtained data of the annual newly diagnosed AIDS cases and AIDS deaths from 1980 to 2014 in MSM population in San Francisco from the Department of Public Health HIV Epidemiology Section. Using the maximum likelihood estimation, we fit the model to the data between 1980 and 1995 to estimate the prior-treatment parameters (see more details in [2]): the recruitment rate of susceptible MSM is $b = 4000$ (95%CI:2295-5705) per year, the disease-induced death rate at the chronic stage for untreated drug-sensitive individuals is $\mu_s^U = 0.28$ (95%CI:0.18-0.39) per year, and the transmission rate in this stage is $\beta_s^U = 0.62$ (95%CI:0.57-0.68) per year. The estimation of the transmission rate is the same as the value of 0.62 shown in [25] for MSM in San Francisco.

The values of other parameters are given as follows. The initial MSM population size is chosen as 69122 [26,27]. The expected duration of a sexual career in San Francisco is assumed to be about 47 years (age 18-65) as in [28]. Thus, we have the removal rate $m = 1/47 = 0.021$ per year. For untreated drug-sensitive individuals, we choose the duration of the primary stage as $a_p = 1.7$ months [29], the duration of the chronic stage as $d_c = 7.5$ years [30], and the duration of the AIDS stage as $d_A = 1.2$ years (12 months to 20 months in [31]). Thus, we obtain the rate of progression to the asymptomatic stage is $\delta_1 = 1/a_p = 1/(1.7/12) = 7.06$ per year. Similarly, we have the rate of progression to the AIDS stage is $\delta_s^U = 1/d_c = 1/7.5 = 0.13$ per year and the disease-induced death rate in the AIDS stage is $\mu_A = 1/d_A = 1/1.2 = 0.83$ per year. We assume the chronic stage d_r for untreated drug-resistant cases is 25% longer than untreated drug-sensitive cases, i.e., $d_r = 1.25d_c = 9.38$ years, then the progression rate to the AIDS stage is $\delta_r^U = 1/d_r = 1/9.38 = 0.11$ per year. The transmission rates in the primary stage and AIDS stage are assumed to be 5.3 and 7 times more infectious than during chronic infection, respectively, i.e., $\beta_s^p = 5.3\beta_s^U, \beta_r^p = 5.3\beta_r^U$ [29] and $\beta_s^A = 7\beta_s^U, \beta_r^A = 7\beta_r^U$ [32] (see Appendix Figures 4-5 in [2]). Here we assume all infected individuals are initially infected with drug-sensitive strains, so there are only susceptible compartment and untreated drug-sensitive individuals at different infection stages at the beginning of the epidemic (1980-1995).

We used the data from 1996 to 2006 (because ART was widely available after 1995 [33]) to estimate treatment-related parameters. The treatment rate is estimated as $\eta = 0.38$ (95%CI:0.08-0.81) per year, i.e., the average time from infection to ART initiation is $a_{ART} = 2.8$ years according to the relationship $\eta = 1/(a_{ART} - a_p)$. The fraction of treated gay men in San Francisco is calculated as $F_s = 46.4\%$ (95%CI:15.3%-64.9%) based on Eq. (3), which is close to the fraction in [3] where about 50% of HIV-infected MSM take ART. The disease-induced death rate in the post-treatment chronic stage is estimated as $\mu_s^T = 0.05$ per year (95%CI:0.01-0.27) for drug-sensitive individuals and $\mu_r^T = 1.75\mu_s^T = 0.088$ per year for drug-resistant individuals [34]. In this fitting process, we chose a bigger recruitment rate $b = 5600$ per year to yield simulated prevalence, total infected individuals and population size simultaneously consistent with the prevalence data (**Figure 2c** in the main text), persons living with HIV/AIDS data and total MSM population size data respectively as closely as possible, which we did not fit directly (see [2] for more details). We also chose the relative transmissibility for treated drug-resistant individuals ($\beta_r^T = 0.2\beta_s^U$, i.e, the baseline second-line drug effectiveness was estimated as 80%) to match the prevalence data of transmitted drug resistance (**Appendix Figure 3**, see [2] for more details) under the assumption that the transmission rate for untreated drug-resistant individuals β_r^U was the average of that for treated drug-resistant β_r^T and untreated drug-sensitive individuals β_s^U based on their relationship

$\beta_s^U > \beta_r^U > \beta_r^T$ [3], i.e., $\beta_r^U = (\beta_r^T + \beta_s^U)/2 = 0.6\beta_s^U$. The fraction of treated cases that are drug resistant is chosen as $f_r = 25\%$ (33% of MSM are virally unsuppressed [28] and of which 76% have drug resistance [35]). The result of the HPTN 052 clinical trial [36, 37] showed that treatment led to 96% reduction in infectivity. Thus, the transmission rate in treated individuals without drug resistance is only 4% as transmissible as HIV positives not receiving ART ($\beta_s^T = 0.04\beta_s^U$).

After 2006, San Francisco had name-based HIV reporting and incorporated monitoring initial primary care visit into standard HIV public health investigation for newly diagnosed cases which improved the treatment rate and shortened the time to entry into HIV medical care [38]. So we used the data from 2006 to 2012 to estimate the growing treatment rate $\eta = 0.7$ per year and earlier ART initiating timing $a_{ART} = 1.6$ years. All the other parameters are fixed in **Appendix Table 1**.

In 2012, the PrEP was approved by FDA and PrEP use has increased to 9.6% in 2014 in San Francisco [39]. We calibrated the model by choosing the PrEP receiving rate as $\xi = 0.06$ per year to match the 9.6% PrEP coverage in 2014 and the coverage will reach 25% in 2023 (low coverage) at this PrEP receiving rate. If we consider the cases that PrEP coverage will reach 50% (medium coverage) or 80% (high coverage) after 5 years (in 2023), then the rate that susceptible receive PrEP should increase to $\xi = 0.20$ or $\xi = 0.68$ per year respectively. We assumed the PrEP effectiveness against drug-sensitive strains was $\epsilon_s = 53\%$ in the base case based on the meta-analysis results in [40] and against the resistant strains was $\epsilon_r = 50\% \times \epsilon_s = 26.5\%$ [13–15]. The rate discontinuing PrEP was chosen as $w = 8\%$ per year based on the data in [41] that approximately 8% of participants gave up PrEP due to side effect concerns and no perceived HIV risk in a one-year project in San Francisco. We considered how the above PrEP scenarios (low, medium, high PrEP coverage) interacted with the implementation of new even earlier ART guidelines (initiation at 1 year post infection averagely), in contrast with previous ART guidelines (initiation at 1.6 years post infection averagely), will affect the cost-effectiveness of PrEP over the next 20 years (from 2018 to 2038).

Next, we derive the relationship between extended life expectancies and infection age (more detail was shown in [2]). It can be seen from [10,11] that suppressed patients (HIV-1 RNA ≤ 400 copies/ml) who had CD4+ count < 200 or ≥ 350 cells/mm^3 at ART start can live mean 30 or 45 years after treatment, respectively. In addition, Fig. 1(B) in [12] shows that CD4+ count decreases with time since infection (infection age). Specifically, CD4+ count < 200 and ≥ 350 cells/mm^3 corresponds to the infection age of 7-9 years and 0-6 years, respectively. We chose the average infection age 8 years and 3 years for CD4+ count < 200 and ≥ 350 cells/mm^3, respectively. Therefore, if an infected individual is treated at 8 years post-infection with viral suppression, then he would live 30 years. However, if the treatment begins at 3 years, he would live 45 years. According to the assumed linear decreasing relationship Eq. (4) between the average duration L_s^T from ART initiation to death for suppressed individuals and ART initiation timing a_{ART} (blue line in Appendix Figure 3a in [2]), we obtain

$$L_s^T - 45 = \frac{30-45}{3-8}(a_{ART} - 3), \text{ i.e., } L_s^T = 54 - 3a_{ART}.$$

This implies that there would be 3 years longer to live if ART had started one year earlier. The unsuppressed individuals (HIV-1 RNA > 400 copies/ml) will take 11 years off life expectancy than treated suppressed individuals [10, 11]. Thus, if an infected individual is treated at 8 and 3 years post-infection without viral suppression, then he can live 19 and 34 years, respectively. Similarly, we have the relationship between the average duration L_r^T from ART initiation to death for unsuppressed individuals and ART initiation timing a_{ART} (red line in

Appendix Figure 3a in [2]) as follows

$$L_r^T - 34 = \frac{19-34}{3-8}(a_{ART} - 3), \text{ i.e., } L_r^T = 43 - 3a_{ART}.$$

When $a_{ART} = 2.8$ before 2006, we have $L_s^T = 45.6$ and $L_r^T = 34.6$. When $a_{ART} = 1.6$ after 2006, we have $L_s^T = 49.2$ and $L_r^T = 38.2$. If $a_{ART} = 1$, we get $L_s^T = 51$ and $L_r^T = 40$. Notice that 76% of treatment-failed patients have resistance to one or more antiretroviral drugs [35]. Therefore, we assume that the above relationships between the extended life expectancy and ART initiating timing for unsuppressed and suppressed patients still hold for treated individuals who do or do not develop drug resistance. See [2] for more details. All the other parameters can be found in **Appendix Table 1**.

5 Supplementary Tables and Figures

5.1 Supplementary Tables

Appendix Table 1 shows the parameters used in simulation. **Appendix Table 2** gives more detail on the costs of expanding PrEP coverage and earlier ART, incremental to status quo (base case).

Appendix Table 1: Parameters and values for simulation and data fitting

Parameter	Description	Baseline or estimated mean value	Source
a_p	The duration of the primary stage	1.7 months	[29]
d_c	The duration of the chronic stage (untreated, drug-sensitive)	7.5 years	[30]
d_r	The duration of the chronic stage (=$1.25d_c$, untreated, drug-resistant)	9.38 years	[2]
d_A	The duration of the AIDS stage	1.2 years	[31]
b	Susceptible population admission rate	4000 per year before 1995 (95%CI:[2295,5705])	Fitted
		5600 per year after 1995	[2]
m	Removal rate due to changes in sexual behavior	0.021 per year	[28]
$N(0)$	Initial total MSM population size	69122	[26, 27]
μ_s^U	The disease-induced death rate at the chronic stage (untreated, drug-sensitive)	0.28 per year (95%CI:[0.18,0.39])	Fitted
μ_A	The disease-induced death rate at the AIDS stage (=$1/d_A$)	0.83 per year	Calculated
δ_1	Rate of progression to the chronic stage (=$1/a_p$)	7.06 per year	Calculated

Appendix Table 1: Parameters and values for simulation and data fitting

Parameter	Description	Baseline or estimated mean value	Source
δ_s^U	Rate of progression to the AIDS stage for untreated drug-sensitive individuals (=$1/d_c$)	0.13 per year	Calculated
δ_r^U	Rate of progression to the AIDS stage for untreated drug-resistant individuals (=$1/d_r$)	0.11 per year	Calculated
β_s^U	The transmission rate at the chronic stage (untreated, drug-sensitive)	0.62 per year (95%CI:[0.57,0.68])	Fitted
β_s^p	The transmission rate at the primary stage (untreated, drug-sensitive)	$5.3\beta_s^U$	[29]
β_s^A	The transmission rate at the AIDS stage (drug-sensitive)	$7\beta_s^U$	[32]
β_s^T	The transmission rate in the post-treatment chronic stage (drug-sensitive)	$0.04\beta_s^U$	[36, 37]
β_r^T	The transmission rate in the post-treatment chronic stage (drug-sensitive)	$0.2\beta_s^U$	[2]
β_r^U	The transmission rate at the chronic stage (=$(\beta_s^U + \beta_r^T)/2$, untreated, drug-resistant)	$0.6\beta_s^U$	[2]
β_r^p	The transmission rate at the primary stage (untreated, drug-resistant)	$5.3\beta_r^U$	[29]
β_r^A	The transmission rate at the AIDS stage (drug-resistant)	$7\beta_r^U$	[32]
η	The treatment rate	0.38 per year before 2006 (95%CI:[0.08,0.81])	[2]
		0.7 per year after 2006 (95%CI:[0.68,0.71])	[2]
a_{ART}	The average time from infection to treatment	2.8 years before 2006<br>1.6 years after 2006	[2]

Appendix Table 1: Parameters and values for simulation and data fitting

Parameter	Description	Baseline or estimated mean value	Source
L_s^T	The post-treatment extended life expectancy for drug-sensitive cases	45.6 years before 2006<br>49.2 years after 2006	[2]
L_r^T	The post-treatment extended life expectancy for drug-resistant cases	34.6 years before 2006<br>38.2 years after 2006	[2]
δ_s^T	Rate of progression to the AIDS stage for treated drug-sensitive individuals (=$1/(L_s^T - d_A)$)	0.022 per year before 2006<br>0.020 per year after 2006	Calculated
δ_r^T	Rate of progression to the AIDS stage for treated drug-resistant individuals (=$1/(L_r^T - d_A)$)	0.029 per year before 2006<br>0.026 per year after 2006	Calculated
μ_s^T	The disease-induced death rate in the post-treatment chronic stage (drug-sensitive)	0.05 per year (95%CI:[0.01,0.27])	Fitted
μ_r^T	The disease-induced death rate in the post-treatment chronic stage (drug-resistant)	$1.75\mu_s^T$	[34]
f_r	The fraction of treated cases that are drug resistant	0.25	[28,35]
ϵ_s	The PrEP effectiveness against drug-sensitive strains	53%	[40]
ϵ_r	The PrEP effectiveness against drug-resistant strains	$0.5\epsilon_s$	[13–15]
Quality-of-life factors			
Q_S	Uninfected, no PrEP	1.00	[16,43–45]
Q_P	Uninfected, receiving PrEP	1.00 (0.90-1.00)	[45]
Q_{s1}^U	Acute HIV (Untreated, drug-sensitive)	0.94 (0.73-0.97)	[16,42–45]
Q_{s2}^U	Asymptomatic HIV (Untreated, drug-sensitive)	0.82 (0.82-0.95)	[16,42–45]
Q_{s3}^U	AIDS (Untreated, drug-sensitive)	0.70 (0.60-0.75)	[16,42–45]
Q_{s1}^T	Asymptomatic HIV (Treated, drug-sensitive)	0.83 (0.82-0.87)	[16,43–45]
Q_{s2}^T	AIDS (Treated, drug-sensitive)	0.82 (0.82-0.87)	[16,43–45]

Appendix Table 1: Parameters and values for simulation and data fitting

Parameter	Description	Baseline or estimated mean value	Source
Q^U_{r1}	Acute HIV (Untreated, drug-resistant)	$0.95Q^U_{s1}$	[18, 19]
Q^U_{r2}	Asymptomatic HIV (Untreated, drug-resistant)	$0.95Q^U_{s2}$	[18, 19]
Q^U_{r3}	AIDS (Untreated, drug-resistant)	$0.95Q^U_{s3}$	[18, 19]
Q^T_{r1}	Asymptomatic HIV (Treated, drug-resistant)	$0.95Q^T_{s1}$	[18, 19]
Q^T_{r2}	AIDS (Treated, drug-resistant)	$0.95Q^T_{s2}$	[18, 19]
Cost parameters (2017 US $)			
c_{neg}	Cost of HIV testing (antibody test) and counseling for uninfected individuals	42 (5-75)	[44, 45]
c_{pos}	Cost of HIV testing (antibody test) and counseling for infected individuals	119 (50-300)	[44, 45]
c_{diag}	Cost of HIV diagnosis	633 (125-1200)	[44, 45]
c_{GT}	Cost of HIV genotype resistance test	218 (54-239)	[19]
c^{ART}_s	Annual cost of first-line drugs	15450 (9170-22300)	[46]
c^{ART}_r	Annual cost of second-line drugs	$1.24c^{ART}_s$	[47]
c_P	Annual cost of PrEP	17874 (7250-22342)	[48]
Annual HIV-related healthcare costs			
c^U_{s1}	Acute HIV (Untreated, drug-sensitive)	37 (10-500)	[44, 45]
c^U_{s2}	Asymptomatic HIV (Untreated, drug-sensitive)	4244 (2460-5950)	[44, 45]
c^U_{s3}	AIDS (Untreated, drug-sensitive)	21260 (10380-33240)	[46]
c^T_{s1}	Asymptomatic drug-sensitive HIV-Treated with first-line drugs (excludes ART costs)	6896 (4190-9280)	[16, 43–45]
c^T_{s2}	AIDS with drug-sensitive strain-Treated with first-line drugs (excludes ART costs)	10609 (4870-17160)	[16, 43–45]

Appendix Table 1: Parameters and values for simulation and data fitting

Parameter	Description	Baseline or estimated mean value	Source
c_{r1}^{U}	Acute HIV (Untreated, drug-resistant)	c_{s1}^{U}	Assumed
c_{r2}^{U}	Asymptomatic HIV (Untreated, drug-resistant)	c_{s2}^{U}	Assumed
c_{r3}^{U}	AIDS (Untreated, drug-resistant)	c_{s3}^{U}	Assumed
c_{r1}^{T}	Asymptomatic drug-resistant HIV-Treated with second-line drugs (excludes ART costs)	c_{s1}^{T}	Assumed
c_{r2}^{T}	AIDS with drug-resistant strain-Treated with second-line drugs (excludes ART costs)	c_{s2}^{T}	Assumed

CI=confidence interval; PrEP=preexposure prophylaxis; ART=antiretroviral therapy.

Appendix Table 2: Costs of expanding PrEP coverage and earlier ART, incremental to status quo (base case).

Strategy	Incremental HIV healthcare costs (million US $)	Incremental PrEP costs (million US $)	Incremental first-line drug costs (million US $)	Incremental second-line drug costs (million US $)	Incremental testing and counselling costs (million US $)	Incremental diagnosis costs (million US $)	Incremental genotype resistance test costs (million US $)	Total Incremental costs (million US $)
Medium PrEP coverage	−148.1[a]	5929	−160.2	−129.2	−7.6	−2.7	−4.5	5477
High PrEP coverage	−266.6	12408	−283.9	−244.5	−16.1	−4.6	−8.2	11584
Low PrEP coverage +Earlier ART	−60.0	266.1	−56.0	43.0	14.7	−0.3	−0.4	207
Medium PrEP coverage +Earlier ART	−168.1	6256.8	−171.8	−76.8	2.1	−2.4	−4.0	5836
High PrEP coverage +Earlier ART	−264.6	12659	−272.4	−190.3	−11.5	−4.1	−7.2	11909

PrEP=preexposure prophylaxis; ART=antiretroviral therapy.

[a] A negative number refers to costs saved compared with the status quo.

5.2 Supplementary Figures

Appendix Figure 1 shows the flow diagram of the model (6). **Appendix Figure 2** illustrates the dynamics of susceptible with PrEP. **Appendix Figure 3** shows how the proportion of new infections that are drug resistant varies with the time.

Appendix Figures 4-7 demonstrate additional sensitivity analyses of our ICER estimates of low or medium PrEP coverage with earlier ART, and medium or high PrEP coverage without earlier ART compared to status quo. The ICER is less sensitive to the effectiveness of PrEP against drug-sensitive strains with earlier ART (**Figure 5** in the main text and **Appendix Figures 6-7**) than without earlier ART (**Appendix Figures 4-5**), because earlier ART substantially increases QALYs and thereby reduces the potential benefits of PrEP. If the relative effectiveness of PrEP against resistant versus susceptible strains decreases from 50% to 0 (PrEP is completely ineffective against resistant strains), the ICER would increase by 16% from $115 320 to $133 660 per QALY gained.

The three drug resistance parameters (the fraction of acquired drug resistance, the costs of second-line drugs, and the quality of life for drug-resistant individuals) had limited effects on cost-effectiveness results because PrEP costs comprise 87% of total costs for high PrEP coverage plus earlier ART (**Table 1** in the main text), and thus drug-resistance related costs only minimally impact total costs. However, second-line drug effectiveness greatly affects the cost-effectiveness of PrEP (**Figure 5** in the main text) with greater effectiveness leading to a higher ICER. Although increasing second-line drug effectiveness increases total QALYs, it causes an even greater increase in total costs (hence a higher ICER) since the consequent reduction in new HIV infections means a larger susceptible population on PrEP. Thus, the reductions (savings) in treatment costs are largely counterbalanced by substantial increases in PrEP costs. Nonetheless, highly effective second-line drugs remain cost-effective.

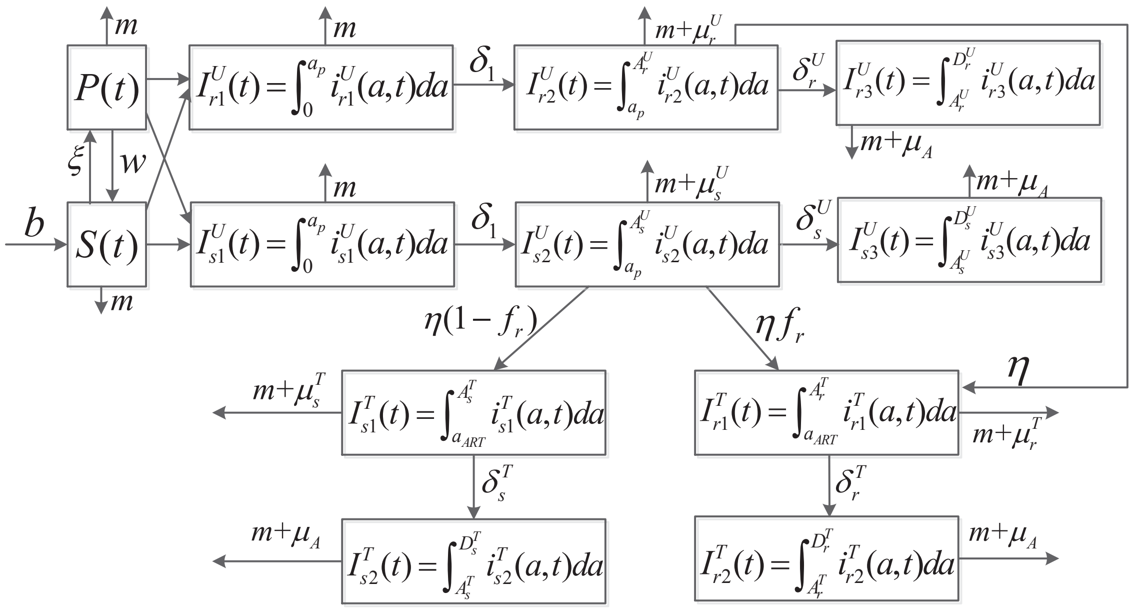

Appendix Figure 1: A full flow diagram illustrating our transmission model of HIV epidemic dynamics with PrEP and ART interventions, accounting for the acquisition of drug resistance following ART and the transmission of drug resistance for model (6). Abbreviation: ART, antiretroviral therapy; PrEP, pre-exposure prophylaxis.

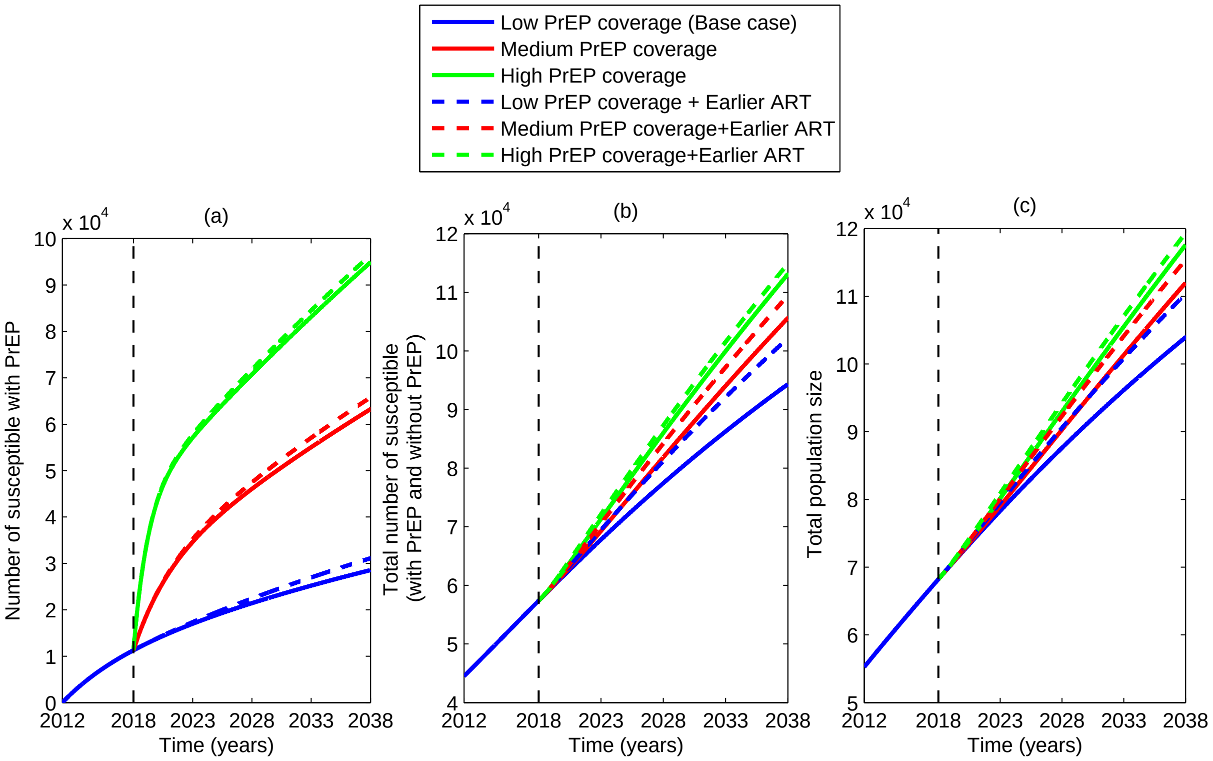

Appendix Figure 2: The number of susceptible with PrEP (a), total susceptible (b), and total population size (c) varied with the time for low, medium, and high PrEP coverage with or without earlier ART. Abbreviation: ART, antiretroviral therapy; PrEP, pre-exposure prophylaxis.

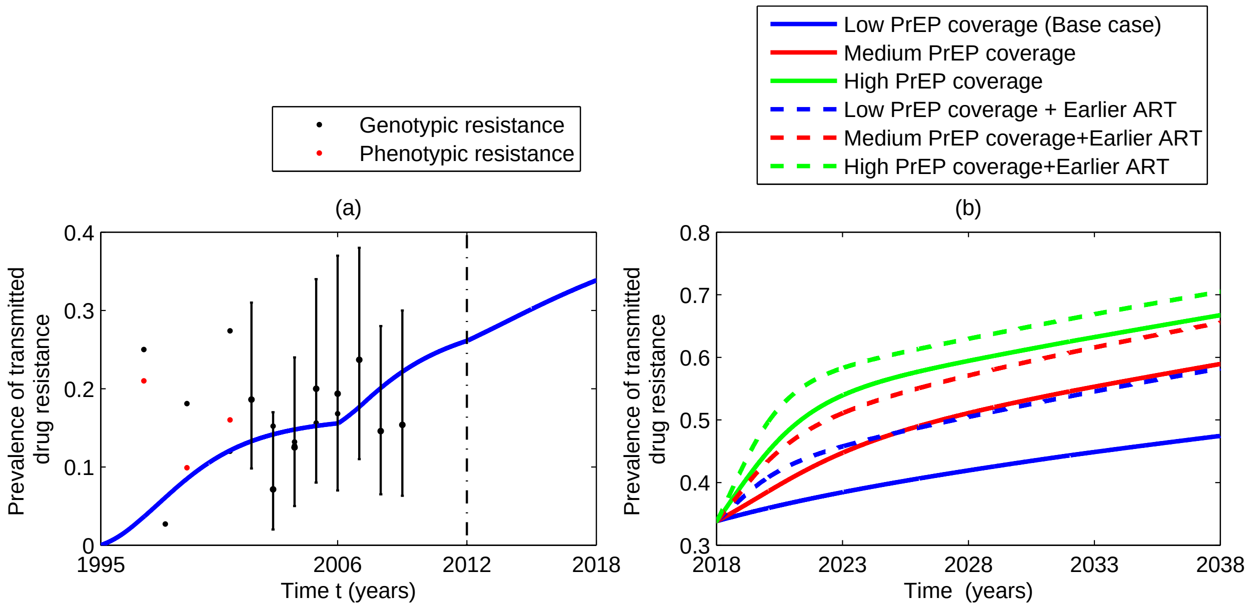

Appendix Figure 3: (a) Observed proportion of new infections that are drug resistant (black dots, with 95% confidence interval if available, denote genotypic resistance and red dots denote phenotypic resistance) among previous cohorts and model fit (blue line). See [2] for more detail. Previous comparison between model and empirical data for trends of percentage of new drug-resistant infections in San Francisco (1996-2005) can be found in [49]. (b) Predicted proportion of new infections that are drug resistant over next 20 years for low, medium, and high PrEP coverage with or without earlier ART. This is in accordance with the results of Figure 4 in [13]. Abbreviation: ART, antiretroviral therapy; PrEP, pre-exposure prophylaxis.

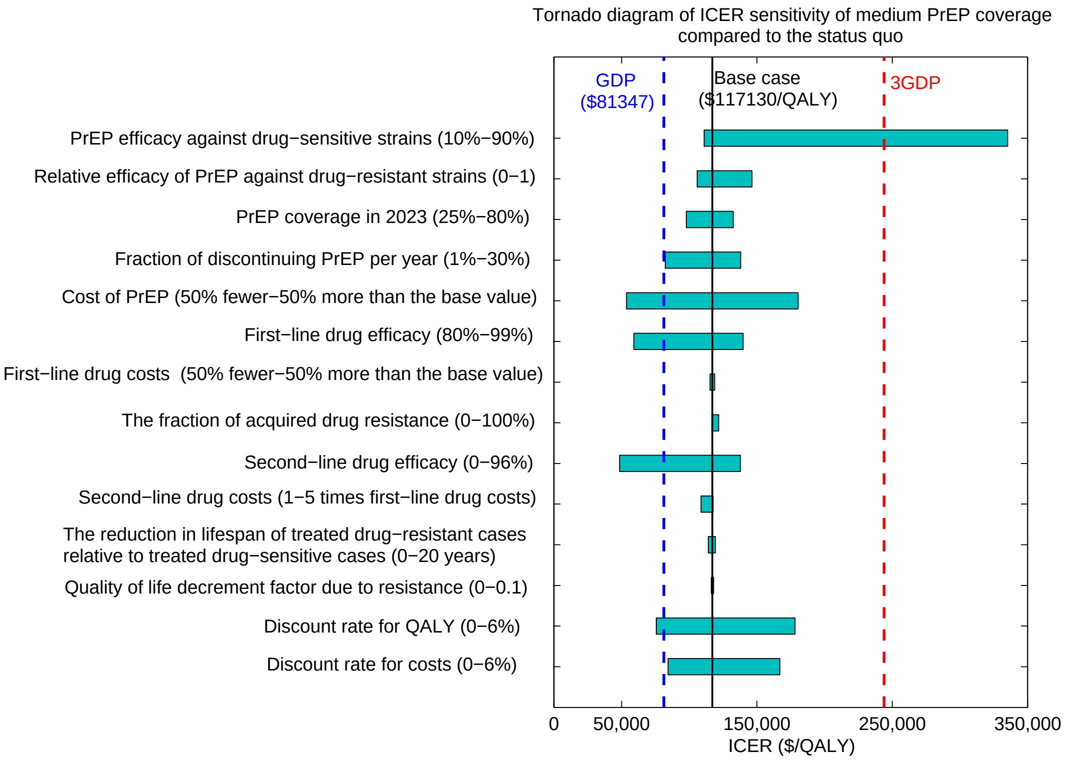

Appendix Figure 4: One-way sensitivity analysis on cost-effectiveness of medium PrEP coverage without earlier ART compared to the status quo. The horizonal bars represent the range of the incremental cost-effectiveness ratios (ICERs) as each variable is varied across its plausible range listed. The solid vertical line indicates the base case ICER ($117 130 per QALY gained). The dashed vertical line represents the per capita gross domestic product (GDP) for San Francisco ($81347 in 2015 [50]), a threshold denoting a very cost-effective use of resources, by international standards [22–24]. Abbreviation: PrEP, pre-exposure prophylaxis; ART, antiretroviral therapy; ICER, incremental cost-effectiveness ratio; QALY, quality-adjusted life-year; GDP, gross domestic product.

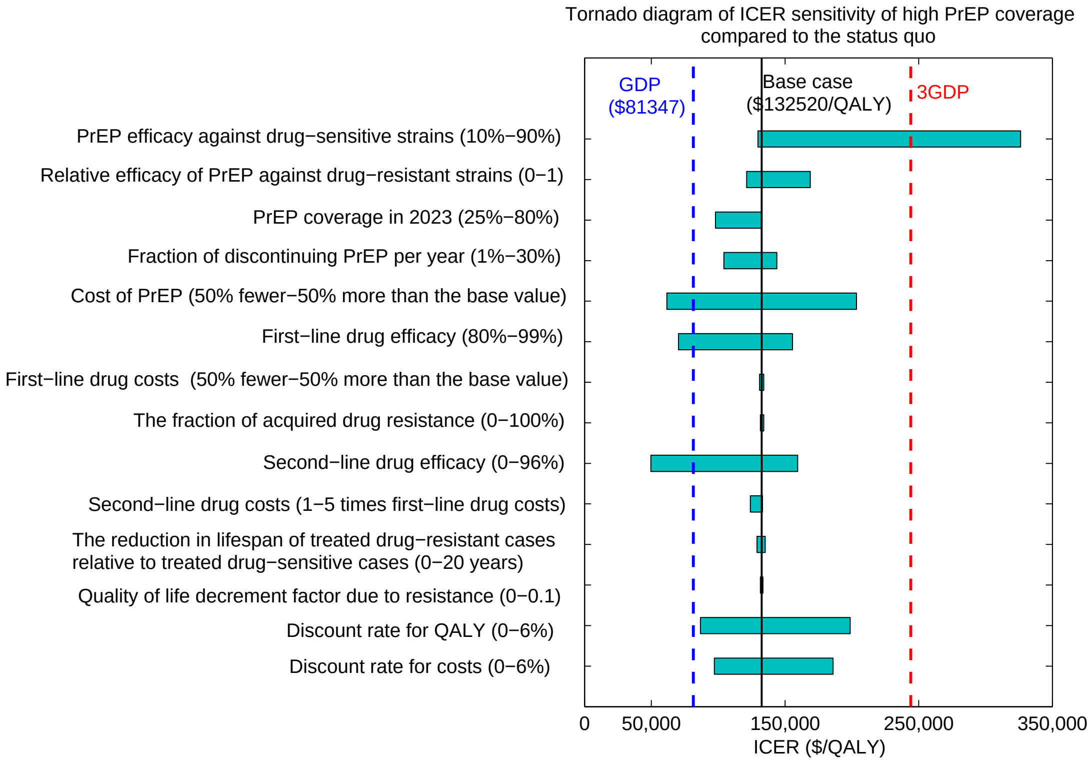

Appendix Figure 5: One-way sensitivity analysis on cost-effectiveness of high PrEP coverage without earlier ART compared to the status quo. The horizonal bars represent the range of the incremental cost-effectiveness ratios (ICERs) as each variable is varied across its plausible range listed. The solid vertical line indicates the base case ICER ($132 520 per QALY gained). The dashed vertical line represents the per capita gross domestic product (GDP) for San Francisco ($81347 in 2015 [50]), a threshold denoting a very cost-effective use of resources, by international standards [22–24]. Abbreviation: PrEP, pre-exposure prophylaxis; ART, antiretroviral therapy; ICER, incremental cost-effectiveness ratio; QALY, quality-adjusted life-year; GDP, gross domestic product.

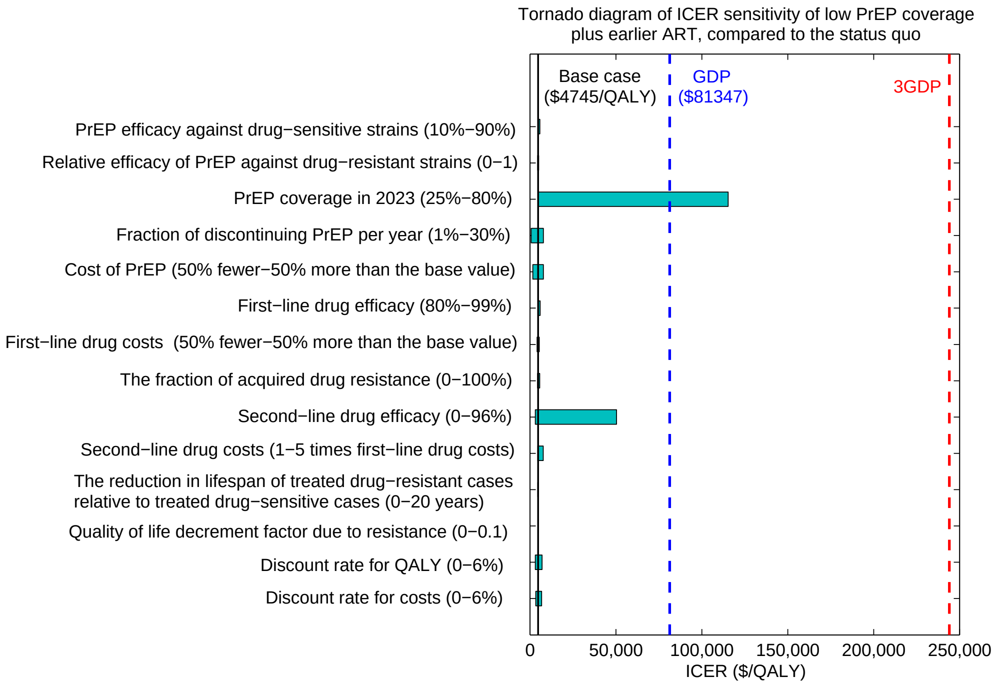

Appendix Figure 6: One-way sensitivity analysis on cost-effectiveness of low PrEP coverage plus earlier ART compared to the status quo. The horizonal bars represent the range of the incremental cost-effectiveness ratios (ICERs) as each variable is varied across its plausible range listed. The solid vertical line indicates the base case ICER ($4745 per QALY gained). The dashed vertical line represents the per capita gross domestic product (GDP) for San Francisco ($81347 in 2015 [50]), a threshold denoting a very cost-effective use of resources, by international standards [22–24]. Abbreviation: PrEP, pre-exposure prophylaxis; ART, antiretroviral therapy; ICER, incremental cost-effectiveness ratio; QALY, quality-adjusted life-year; GDP, gross domestic product.

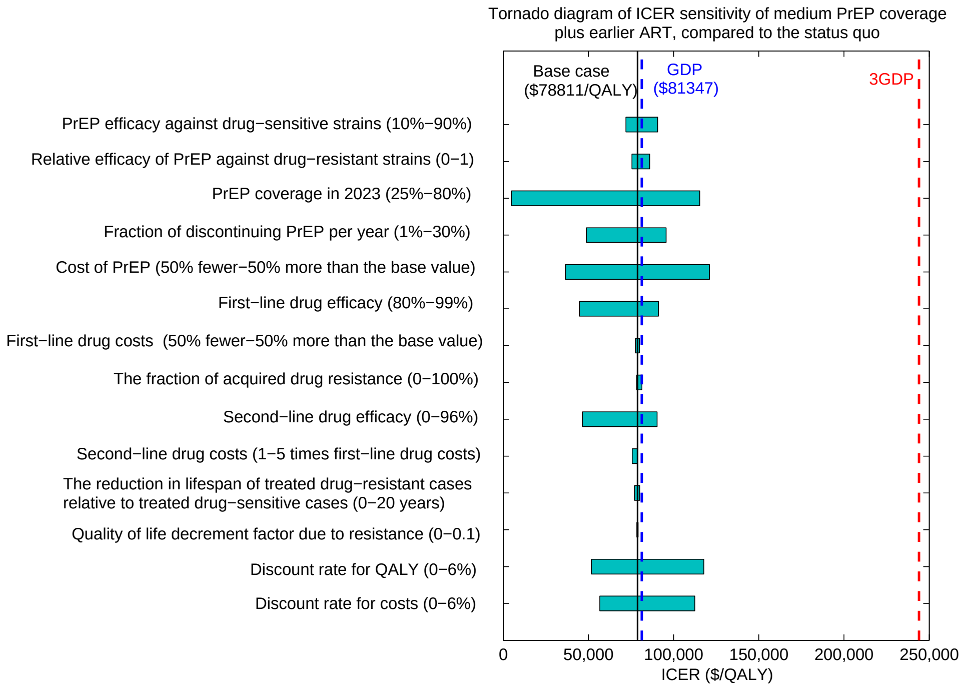

Appendix Figure 7: One-way sensitivity analysis on cost-effectiveness of medium PrEP coverage plus earlier ART compared to the status quo. The horizonal bars represent the range of the incremental cost-effectiveness ratios (ICERs) as each variable is varied across its plausible range listed. The solid vertical line indicates the base case ICER ($78 811 per QALY gained). The dashed vertical line represents the per capita gross domestic product (GDP) for San Francisco ($81347 in 2015 [50]), a threshold denoting a very cost-effective use of resources, by international standards [22–24]. Abbreviation: PrEP, pre-exposure prophylaxis; ART, antiretroviral therapy; ICER, incremental cost-effectiveness ratio; QALY, quality-adjusted life-year; GDP, gross domestic product.

References

[1] Shen MW, Xiao YN, Rong LB. Global stability of an infection-age structured HIV-1 model linking within-host and between-host dynamics. Math Biosci **2015**; 263:37-50.

[2] Shen MW, Xiao YN, Rong LB, Meyers LA, Bellan SE. Early antiretroviral therapy and potent second-line drugs could decrease HIV incidence of drug resistance. P Roy Soc B-Biol Sci **2017**; 284: 20170525.

[3] Blower SM, Gershengorn HB, Grant RM. A tale of two futures: HIV and antiretroviral therapy in San Francisco. Science **2000**; 287:650-4.

[4] Fraser C, Hollingsworth TD, Chapman R, de Wolf F, Hanage WP. Variation in HIV-1 set-point viral load: epidemiological analysis and an evolutionary hypothesis. Proc Natl Acad Sci USA **2007**; 104:17441-6.

[5] Dodd PJ, Garnett GP, Hallett TB. Examining the promise of HIV elimination by 'test and treat' in hyperendmic settings. AIDS **2010**; 24:729-35.

[6] Nagelkerke NJD, Jha P, de Vlas SJ, et al. Modelling HIV/AIDS epidemics in Botswana and India: impact of interventions to prevent transmission. Bull World Health Organ **2002**; 80:89-96.

[7] Ramadhani HO, Bartlett JA, Thielman NM, et al. Association of first-line and second-line antiretroviral therapy adherence. Open Forum Infect Dis **2014**; 1(2):ofu079.

[8] May M, Gompels M, Delpech V, et al. Impact of late diagnosis and treatment on life expectancy in people with HIV-1: UK Collaborative HIV Cohort (UK CHIC) Study. BMJ **2011**; 343:d6016.

[9] Samji H, Cescon A, Hogg RS, et al. Closing the gap: increases in life expectancy among treated HIV-positive individuals in the United States and Canada. PLoS One **2013**; 8:e81355.

[10] May M, Gompels M, Sabin C. Life expectancy of HIV-1-positive individuals approaches normal, conditional on response to antiretroviral therapy: UK collaborative HIV cohort study. J Int AIDS Soc. **2012**; 15(Suppl 4):18078.

[11] May M, Gompels M, Delpech V, et al. Impact on life expectancy of HIV-1 positive individuals of CD4+ cell count and viral load response to antiretroviral therapy. AIDS **2014**; 28:1193-202.

[12] Williams BG, Dye C. Antiretroviral drugs for tuberculosis control in the era of HIV/AIDS. Science **2003**; 301:1535-7.

[13] Supervie V, Garca-Lerma JG, Heneine W, Blower S. HIV, transmitted drug resistance, and the paradox of preexposure prophylaxis. Proc Natl Acad Sci USA **2010**; 107:12381-6.

[14] Supervie V, Barrett M, Kahn JS, et al. Modeling dynamic interactions between pre-exposure prophylaxis interventions & treatment programs: predicting HIV transmission & resistance. Sci Rep. **2011**; 1:185.

[15] Abbas UL, Anderson RM, Mellors JW. Potential impact of antiretroviral chemoprophylaxis on HIV-1 transmission in resource-limited settings. PLoS One. **2007**; 2:e875.

[16] Long EF, Brandeau ML, Owens DK. The cost-effectiveness and population outcomes of expanded HIV screening and antiretroviral treatment in the United States. Ann Intern Med **2010**; 153:778-89.

[17] Li J, Gilmour S, Zhang H, Koyanagi A, Shibuya K. The epidemiological impact and cost-effectiveness of HIV testing, antiretroviral treatment and harm reduction programs. AIDS **2012**; 26:2069-78.

[18] Nichols BE, Sigaloff KC, Kityo C, et al. Increasing the use of second-line therapy is a cost-effective approach to prevent the spread of drug-resistant HIV: a mathematical modelling study. J Int AIDS Soc **2014**; 17:19164.

[19] Drabo EF, Hay JW, Vardavas R, Wagner ZR, Sood N. A cost-effectiveness analysis of preexposure prophylaxis for the prevention of HIV among Los Angeles County men who have sex with men. Clin Infect Dis **2016**; 1-10.

[20] Gold MR, Siegel JE, Russell LB, Weinstein MC. Cost-Effectiveness in Health and Medicine. New York: Oxford University Press, **1996**.

[21] Drummond M, Sculpher M, Torrance G, O'Brien B, Stoddart G. Methods for the economic evaluation of health care programmes. New York: Oxford University Press, **2005**.

[22] World Health Organization. Macroeconomics and health: investing in health for economic development. Report of the Commission on Macroeconomics and Health. Available at: `http://apps.who.int/iris/bitstream/10665/42435/1/924154550X.pdf`. Accessed 5 November 2016.

[23] World Health Organization. The world health report 2002-reducing risks, promoting healthy life. Available at: `http://www.who.int/whr/2002/en/`. Accessed 5 November 2016.

[24] World Health Organization. Making choices in health: WHO guide to cost-effectiveness analysis. Available at: `http://www.who.int/entity/choice/order_form_WHO_Guide.pdf?ua=1`. Accessed 5 November 2016.

[25] McLean AR, Blower SM. Imperfect vaccines and herd immunity to HIV. Proc R Soc Lond Series B **1993**; 253:9-13.

[26] Research and Decisions Corporation. Designing an Effective AIDS Prevention Campaign Strategy for San Francisco: Results From the First Probability Sample of an Urban Gay Male Community, Research and Decisions Corp. **1984**. San Francisco.

[27] Lemp GF, Payne SF, Rutherford GW, et al. Projections of AIDS morbidity and mortality in San Francisco. JAMA **1990**; 263:1497-501.

[28] San Francisco Department of Public Health Population Health Division HIV Epidemiology Section. HIV Epidemiology Annual Repor, **2015**. Available at: `https://www.sfdph.org/dph/files/reports/RptsHIVAIDS/AnnualReport2015-20160831.pdf`. Accessed 5 November 2016.

[29] Bellan SE, Dushoff J, Galvani AP, Meyers LA. Reassessment of HIV-1 Acute Phase Infectivity: Accounting for Heterogeneity and Study Design with Simulated Cohorts. PLoS Med **2015**; 12:e1001801.

[30] Wagner BG, Blower S. Universal access to HIV treatment versus universal 'test and treat': Transmission, drug resistance & treatment costs, Plos One **2012**; 7:e41212.

[31] Jaffar S, Grant AD, Whitworth J, Smith PG, Whittle H. The natural history of HIV-1 and HIV-2 infections in adults in Africa: a literature review. Bull World Health Organ **2004**; 82:462-9.

[32] Hollingsworth TD, Anderson RM, Fraser C. HIV-1 transmission, by stage of infection. J Infect Dis **2008**; 198:687-93.

[33] Katz MH, Schwarcz SK, Kellogg TA, et al. Impact of highly active antiretroviral treatment on HIV seroincidence among men who have sex with men: San Francisco. Am J Public Health **2002**; 92:388-94.

[34] Hogg RS, Bangsberg DR, Lima VD, et al. Emergence of drug resistance is associated with an increased risk of death among patients first starting HAART. PLoS Med **2006**; 3:e356.

[35] Richman DD, Morton SC, Wrin T, et al. The prevalence of antiretroviral drug resistance in the United States. AIDS **2004**; 18:1393-401.

[36] Cohen MS, Chen YQ, McCauley M, et al. Prevention of HIV-1 infection with early antiretroviral therapy. N Engl J Med **2011**; 365:493-505.

[37] Cohen MS, McCauley M, Gamble TR. HIV treatment as prevention and HPTN 052. Curr Opin HIV AIDS **2012**; 7:99-105.

[38] Zetola NM, Bernstein K, Ahrens K, et al. Using surveillance data to monitor entry into care of newly diagnosed HIV-infected persons: San Francisco, 2006-2007. BMC Public Health **2009**; 9:17.

[39] Chen YH, Snowden JM, McFarland W, Raymond HF. Pre-exposure prophylaxis (PrEP) use, seroadaptation, and sexual behavior among men who have sex with men, San Francisco, 2004-2014. AIDS Behav **2016**; 1-7.

[40] Jiang J, Yang X, Ye L, et al. Pre-exposure prophylaxis for the prevention of HIV infection in high risk populations: a meta-analysis of randomized controlled trials. PLoS One **2014**; 9:e87674.

[41] Liu A, Cohen S, Follansbee S, et al. Early experiences implementing preexposure prophylaxis (PrEP) for HIV prevention in San Francisco. PLoS Med **2014**; 11:e1001613.

[42] Tengs TO, Lin TH. A meta-analysis of utility estimates for HIV/AIDS. Med Decis Making **2002**; 22:475-81.

[43] Long EF, Brandeau ML, Owens DK. Potential population health outcomes and expenditures of HIV vaccination strategies in the United States. Vaccine **2009**; 27:5402-10.

[44] Juusola JL, Brandeau ML, Long EF, Owens DK, Bendavid E. The costeffectiveness of symptom-based testing and routine screening for acute HIV infection in men who have sex with men in the USA. AIDS **2011**; 25:1779-87.

[45] Juusola JL, Brandeau ML, Owens DK, Bendavid E. The cost-effectiveness of preexposure prophylaxis for HIV prevention in the United States in men who have sex with men. Ann Intern Med. **2012**; 156:541-50.

[46] Bernard CL, Brandeau ML, Humphreys K, et al. Cost-effectiveness of HIV preexposure prophylaxis for people who inject drugs in the United States. Ann Intern Med **2016**; 165:10-9.

[47] Solem CT, Snedecor SJ, Khachatryan A, et al. Cost of treatment in a US commercially insured, HIV-1-infected population. PLoS One **2014**; 9:e98152.

[48] Horberg M, Raymond B. Financial policy issues for HIV pre-exposure prophylaxis: cost and access to insurance. Am J Prev Med **2013**; 44(Suppl 2):S125-8.

[49] Blower SM, Aschenbach AN, Kahn JO. Predicting the transmission of drug-resistant HIV:comparing theory with data. Lancet Infect Dis **2003**; 3:10-1.

[50] Bureau of Economic Analysis US Department of Commerce. Per capita real GDP by metropolitan area. San Francisco-Oakland-Hayward, CA (Metropolitan Statistical Area). Available at: `http://www.bea.gov/iTable/iTable.cfm?reqid=70&step=1&isuri=1&acrdn=2#reqid=70&step=10&isuri=1&7003=1000&7035=-1&7004=naics&7005=1&7006=41860&7036=-1&7001=21000&7002=2&7090=70&7007=2015&7093=levels` Accessed 5 November 2016.
